# Functional Diversification after Gene Duplication: Paralog Specific Regions of Structural Disorder and Phosphorylation in p53, p63, and p73

**DOI:** 10.1371/journal.pone.0151961

**Published:** 2016-03-22

**Authors:** Helena G. dos Santos, Janelle Nunez-Castilla, Jessica Siltberg-Liberles

**Affiliations:** Department of Biological Sciences, Biomolecular Sciences Institute, Florida International University, Miami, Florida, United States of America; University of Saarland Medical School, GERMANY

## Abstract

Conformational and functional flexibility promote protein evolvability. High evolvability allows related proteins to functionally diverge and perhaps to neostructuralize. p53 is a multifunctional protein frequently referred to as the Guardian of the Genome–a hub for e.g. incoming and outgoing signals in apoptosis and DNA repair. p53 has been found to be structurally disordered, an extreme form of conformational flexibility. Here, p53, and its paralogs p63 and p73, were studied for further insights into the evolutionary dynamics of structural disorder, secondary structure, and phosphorylation. This study is focused on the post gene duplication phase for the p53 family in vertebrates, but also visits the origin of the protein family and the early domain loss and gain events. Functional divergence, measured by rapid evolutionary dynamics of protein domains, structural properties, and phosphorylation propensity, is inferred across vertebrate p53 proteins, in p63 and p73 from fish, and between the three paralogs. In particular, structurally disordered regions are redistributed among paralogs, but within clades redistribution of structural disorder also appears to be an ongoing process. Despite its deemed importance as the Guardian of the Genome, p53 is indeed a protein with high evolvability as seen not only in rearranged structural disorder, but also in fluctuating domain sequence signatures among lineages.

## Introduction

Proteins are dynamic, with a natural tendency to rearrange their conformational ensembles in response to the local environment [1]. Conformational flexibility is associated with functional promiscuity and together they promote evolvability [2]. Evolvability offers a route to functional and structural divergence among related proteins, allowing related proteins to functionally diversify and perhaps to neostructuralize [3] and could manifest as a fold transition, a domain change, or a change in conformational flexibility. Conformational flexibility is enabled through the interplay between amino acid residues in proteins and the degree of flexibility depends on the nature of the amino acids. Similarly, structurally disordered protein regions are conformationally flexible. It follows that if the property of structural disorder is not evolutionarily conserved for homologous sites in a protein family, conformational and functional divergence may be inferred.

Recognized as the Guardian of the Genome, yet infamous for its frequent implication in cancer; p53 is a versatile protein, known to perform numerous functions from DNA binding as a transcription factor to a regulator of apoptosis and beyond [4]. With potential to interact with multiple proteins, p53 has been coined a hub, forming an epicenter of incoming and outgoing signals, such as post-translational modifications and interactions with other biomolecules [5]. Conformational flexibility enables p53 to form specific interactions in a regulated fashion [6]. Consequently, a majority of p53’s interactions are mediated through structurally disordered regions, which are often enriched in post-translational modifications regulating biomolecular interactions, and p53 is no exception [7]. Many of the structurally disordered regions transition to order upon binding [7], while others may endure a shift in the population of the p53 conformational ensemble [8]. Not only is structural disorder essential for p53’s broad functionality, it is accompanied by a complex fitness equation to be considered for every amino acid substitution in this protein. It was recently reported that the structurally disordered regions in the p53 family were highly diversified in amino acid sequence [9].

For every amino acid substitution, the conformational and functional ensemble may be altered, with plausible scenarios ranging from no change to gain-or-loss of function. While globular protein domains must fold to function, structurally disordered regions may be less constrained, challenging the common concept of structure being more conserved than sequence. Many possibilities to balance the fitness equation exist if some functions are benefitted and others slightly impaired. This could result in an expanded nearly-neutral network that would allow rapid sequence divergence [10]. However, for a protein with many extremely important functions, fragility may narrow the nearly-neutral network ultimately resulting in slow sequence divergence [11]. When a multifunctional, structurally disordered protein like p53 accumulates substitutions on evolutionary time scales, does its functional ensemble diverge? The complexity of this question is apparent; structurally disordered proteins are frequently not found to have their complete structural ensemble experimentally determined, and changes in multifunctionality, as seen for a protein hub, are difficult to conclusively deduce experimentally on evolutionary time scales. Here, we take an evolutionary approach informed by linear predictions to investigate the evolutionary dynamics of structural disorder, secondary structure, functional domains, and phosphorylation, in addition to amino acid substitutions, to gain further insights into the functional ensemble and its potential divergence in the p53 family.

## Results

### Origins

**Fig 1:**
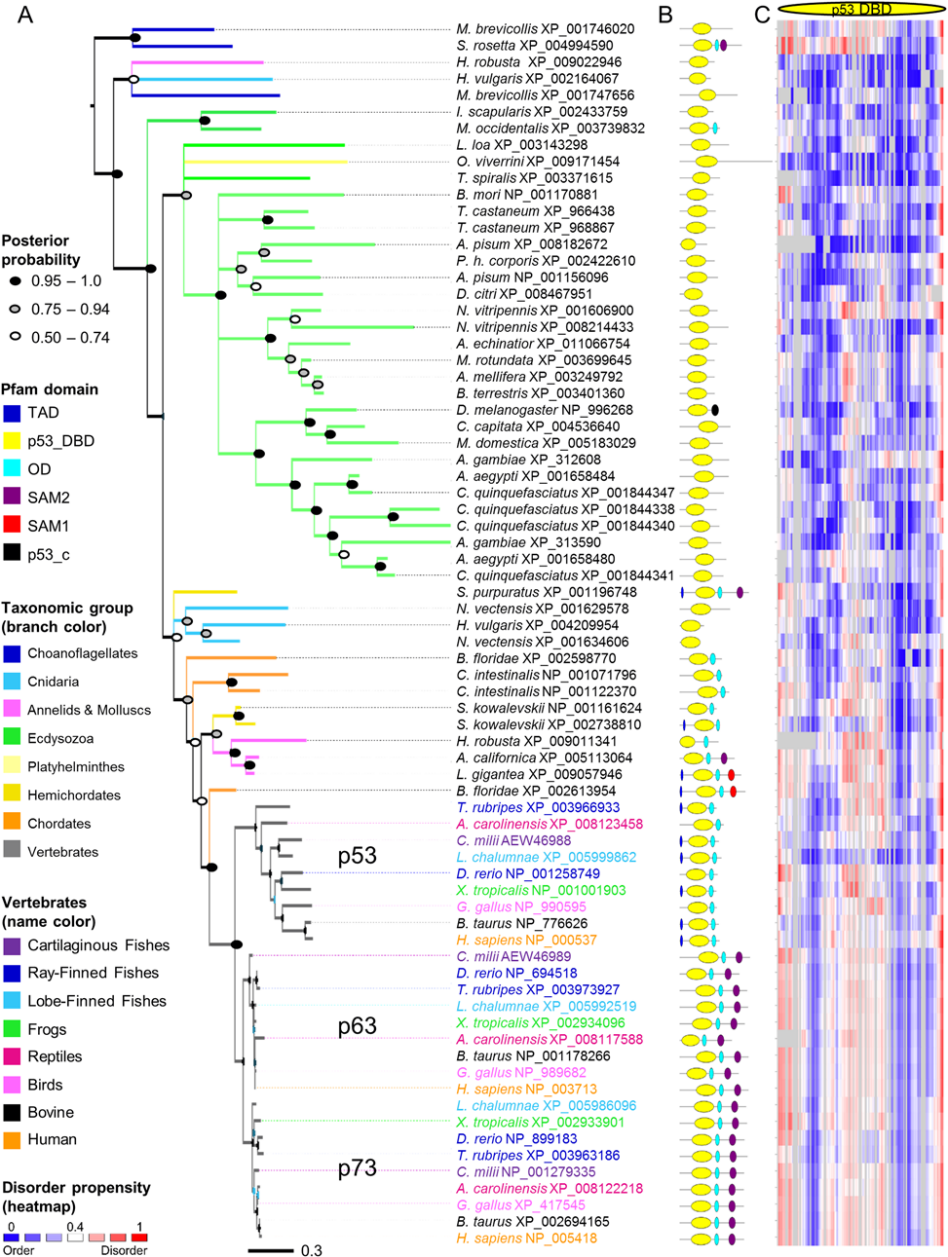
p53 Origins. (A) Overview of the p53 family phylogeny including 74 representative species across Metazoa and in choanoflagellates, built based on their p53 DBD domains. For the invertebrate part of the tree, support values at the nodes indicate posterior probabilities. Nodes with posterior probability <0.5 are unresolved. For detailed support values and for the vertebrate clade, see supplementary material (S1 Fig). (B) Pfam domain architectures showing the multidomain context in which the p53 DBDs are found. (C) Heat map representation of the disorder propensities predicted by IUPred [15] based on the full-length proteins. Rows correspond to protein sequences and columns to alignment sites; the color gradient from blue to white to red mirrors the disorder propensity gradient from low (blue) to high (red), with white being the boundary between order and disorder (alignment gaps are colored in grey).

Reported sightings of a p53 protein and perhaps even a p63/p73 protein in choanoflagellates and invertebrates, suggest that the evolutionary record of p53 predates the beginning of the animal lineage, Metazoa [12]. Thus, a representative p53 family phylogeny including a selection of species ranging from choanoflagellates to primates was constructed for the p53 DNA binding domain (p53 DBD) (Fig 1A). The phylogeny confirms that proteins containing the p53 DBD are found across Metazoa and in choanoflagellates (Fig 1A). In addition to p53 DBD, choanoflagellates and annelids also contain oligomerization domains (ODs) and Sterile Alpha Motif domains (SAMs), while molluscs contain the transactivation domain (TAD), p53 DBD, OD, and SAM. Considering that the same four domain combination is recovered in early chordates, this indicates that this four domain cassette was present prior to the emergence of Ecdysozoa including arthropods (Fig 1B). In the ecdysozoan lineage the p53 ancestor has rapidly diverged and at times regions have been lost, resulting in weak or obliterated traces of the other domains. In hemichordates and early chordates, p53 DBD is found in combinations with OD, TAD and/or SAM. Generally, in non-vertebrates, proteins that not only contain the p53 DBD but additional parts of the four domain cassette tend to cluster, suggesting that more conserved functional sequence motifs may indeed remain within their p53 DBD, compared to the others. Further, cnidarian clusters with the multidomain proteins suggesting that they too may have more of the original functionality left. Noteworthy is that the annelid and mollusc clade, containing *L*. *gigantea* that comprises the four domain cassette, fall inside the hemichordate and early chordate group. *B*. *floridae* has two copies; one (XP_002598770) has the p53 DBD and OD and falls far from all vertebrate p53 domains in this phylogeny, the other (XP_002613954) has the entire four domain cassette. This four domain cassette protein forms the closest outgroup to the entire vertebrate p53 family in this phylogeny and is considered the last common ancestor of all p53, p63 and p73 proteins in vertebrates, in agreement with taxonomy and previous studies [13,14]. In vertebrates, the p53 family consists of two primary clades: one has all p53 proteins, and the other is further split into the p63 and the p73 clades, indicating that p63 and p73 are more similar to each other than to p53.

### Vertebrate expansion

The gene duplication pattern resulting in three vertebrate proteins from one ancestral protein is consistent with two whole genome duplications that supposedly occurred at the time of early vertebrates, after the divergence of *B*. *floridae* but before sharks diverged [13]. To further study the p53 family in vertebrates, a larger vertebrate specific phylogeny was reconstructed. This phylogeny was based on a full-length alignment of 301 sequences with 101, 102, and 98 sequences per p53, p63, and p73 clade, respectively (S2 Fig). The phylogeny shows three specific clades, in agreement with the invertebrate/vertebrate p53 DBD domain tree. Indeed, most vertebrate genomes, from shark to man, seem to encode three genes that belong to the p53 protein family [16], but there are exceptions. Notably, p53 is missing from most of the avian genomes (further discussed below). In addition, there are some lineage-specific small scale duplications of p53. Compared to the ancestral p53 family protein from *B*. *floridae*, all vertebrate proteins in the p53 family have lost domains, but no domains have been added. Proteins in the p63 and p73 clades overall share the three domain composition of p53 DBD, OD, and SAM. TAD is not identified by Pfam (S3 Fig). In the p53 clade, the evolutionary dynamics of TAD is high. TAD is present in shark, but missing from several ray-finned fish, present in lobe-finned fish and snakes, missing in alligators and birds, and present in most mammals (S3 Fig). For the proteins that lack TAD, the sequence may remain but the TAD signature is vague. All p53 proteins lack SAM, thus, it was likely lost before sharks diverged. Rarely, SAM is lost from p63 (*P*. *sinensis* and *B*. *mutus*) or p73 (*U*. *maritimus*), and OD is not found in two sequences in the p53 clade. One is after a lineage-specific duplication in *E*. *edwardii* and the second is from the only bird representative found in data derived from bird genome data, *P*. *humilis*. Lastly, the N-terminus and linkers between domains are variable in length, and in some cases linkers are even absent.

Birds are not well represented in the p53 clade. Only two bird p53 sequences could be found despite extensive efforts. Notably, the sequence for p53 from *G*. *gallus* [17] is not found in its whole genome sequence [18,19]. The only avian genome that has remnants of p53 is *P*. *humilis* [20], although this p53-like sequence only encodes the p53 DBD. *G*. *gallus* p53 has the p53 DBD and the OD but like many other reptiles, it lacks TAD. Further, these two bird sequences fall outside the reptilian clade as the outgroup to mammals and thus, we cannot conclude that these are the main p53 proteins in *P*. *humilis* or *G*. *gallus*. However, given that *G*. *gallus* and *P*. *humilis* are distantly related birds and that they fall close to their expected location in the p53 family phylogeny (S2 Fig), it seems plausible that other bird genomes should still encode at least a p53-like protein, but sequencing it from avian genomes appears challenging.

Domain losses or gains between related proteins are strong indications of functional divergence. A domain loss can occur if the sequence diverges beyond recognition or if the region is physically lost [21]. A domain (and a linker) can also appear lost, if different isoforms or partial sequences are considered. Over time, the domain composition of the p53 family has been altered, with high rate of domain loss in Ecdysozoa where many p53 DBD containing proteins are too short to contain the other domains, but some also have highly divergent OD and SAM domains that no longer generate a significant Pfam domain prediction. In early vertebrates, an ancestral four domain cassette protein was duplicated and subdivided into different proteins, p53, p63, and p73. The p53 clade lost SAM and experienced rapid change in the TAD signature sequence. The p63/p73 clade appears to not change in its current domain organization, but the sequence that once encoded the TAD domain (and may still be present in p63 and p73) has faded beyond recognition, probably prior to the duplication that yielded p63 and p73. Thus, it is possible that a subfunctionalization event followed the first duplication; p53 got most of the TAD domain function, while the p63/p73 ancestor kept the SAM domain.

### Sequence divergence: Rate changes at homologous sites

Following the gene duplication resulting in p63 and p73, p63 is much more constrained, manifested by highly conserved sequences among different species, while the p73 clade is less conserved in sequence. The phylogenies based on full-length protein sequence alignments and their corresponding nucleotide sequence alignment reveal that the rate of sequence divergence is greater in the p53 clade (S2 Fig).

A pairwise comparison (based on the full-length protein alignment) between human and shark sequences in the p53, p63, and p73 clades respectively reveal 51.55%, 76.13% and 76.65% sequence identity. Consequently, p63 and p73 are more similar, with 61.81% sequence identity when comparing shark sequences and 59.24% sequence identity when comparing the human sequences. Further, pairwise sequence identity for shark p53 vs. shark p63 and shark p73, reveal 49.02% and 49.86% respectively. Interestingly, the same comparisons made with the human proteins, p53 vs. p63 and p73, reveal 40.99% and 42.82% pairwise sequence identity, respectively. In summary, the shark p53 family proteins have diverged less than the human counterparts, in accordance with the significantly slower divergence rate found in sharks compared to other vertebrates [22].

### Evolutionary dynamics of structural disorder

**Fig 2:**
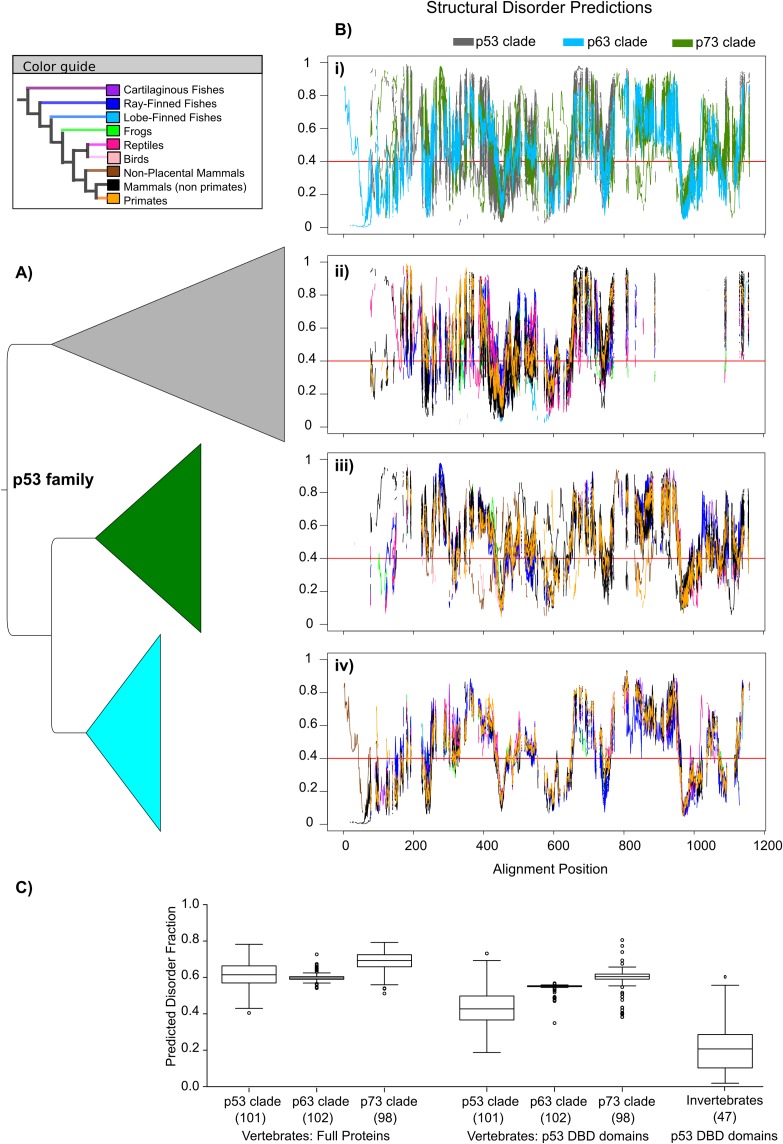
Disorder propensity across the p53 family in vertebrates. (A) Cartoon representation of the p53 family DNA-based phylogeny is shown (p53 clade, grey; p63 clade, blue; p73 clade, green). The p53, p63, and p73 clades contain 101, 102, and 98 sequences, respectively, ranging from shark to human. Horizontal width represents sequence divergence. (B) The profiles of disorder propensity predicted by IUPred [15] are plotted per site according to the multiple sequence alignment. Profiles colored by clade (i) and by species according to the color guide for sequences in the p53 clade (ii), p73 clade (iii), and p63 clade (iv). The cut-off applied to assign structural disorder (≥0.4) or order (<0.4) is marked by the red line. (C) Boxplots showing the fraction of predicted structural disorder for the 301 vertebrate proteins and for the p53 DBD domain for the same vertebrates and for 47 invertebrates separately (all differences in means are statistically significant based on non-parametric tests with p-values <0.05 with the exception of p53-p63 disorder fractions in full length proteins where p-value = 0.25).

Highly dependent on conformational flexibility, the proteins in the p53 family are known to vary in stability; p63 is more stable than p73 and the least stable is p53 [23]. Limited studies of p53 proteins from different species show variation in levels of stability also within the p53 clade. Here, we predicted structural disorder propensity as an approximation for conformational flexibility. The disorder profile for the entire p53 family reveals that the predicted disorder propensity per site is highly variable across the entire length of the protein (Fig 2). Dividing the p53 family into the p53, p63, and p73 clades, reveals that the p63 protein is conserved in disorder propensity across the entire protein, while p53 and p73 show multiple regions with varying disorder propensities across their clades (Fig 2). Classifying the sites into either disorder (if the structural disorder propensity is ≥0.4) or order (if the structural disorder propensity is <0.4), reveals that, on average, predicted disorder fractions per protein are similar in p53 and p63 clades and higher in p73 clade (means: 0.62 and 0.60 and 0.69, with standard deviations: 0.07, 0.03 and 0.05, respectively). Proteins in the p53 clade show a broader range of disorder, ranging from 0.40 to 0.78 (Fig 2C). However, since p53 has a different domain composition than p63 and p73, comparing only the DBD offers further insights. DBDs in the p53 clade are, on average, predicted to be more ordered than the DBDs in p63 and p73, with p73 being more disordered than p63 (Fig 2C). The mean and standard deviations are 0.43 (s.d. 0.09), 0.54 (s.d. 0.03) and 0.58 (s.d. 0.08) in p53, p63 and p73 clades (differences in means between them are significant based on non-parametric tests with p-values <0.05). In the p63 and p73 clades, a decrease in the fraction of disorder in DBD domains in ray-finned fish can be observed (S4 Fig). On the contrary, the p53 clade shows the opposite trend, with many ray-finned fish being among the most disordered. It should also be noted that the lobe-finned fish *L*. *chalumnae* have the most ordered DBD among the entire vertebrate p53 family (S4 Fig). However, also considering the invertebrate p53 DBD, the fractions of disorder in the p53 DBDs are on average smaller than in vertebrates but also more variable within the group (mean 0.23, s.d. 0.16). Single-domain proteins are predicted to be more ordered than those that have contained more of the four domain cassette (S5 Fig).

**Fig 3:**
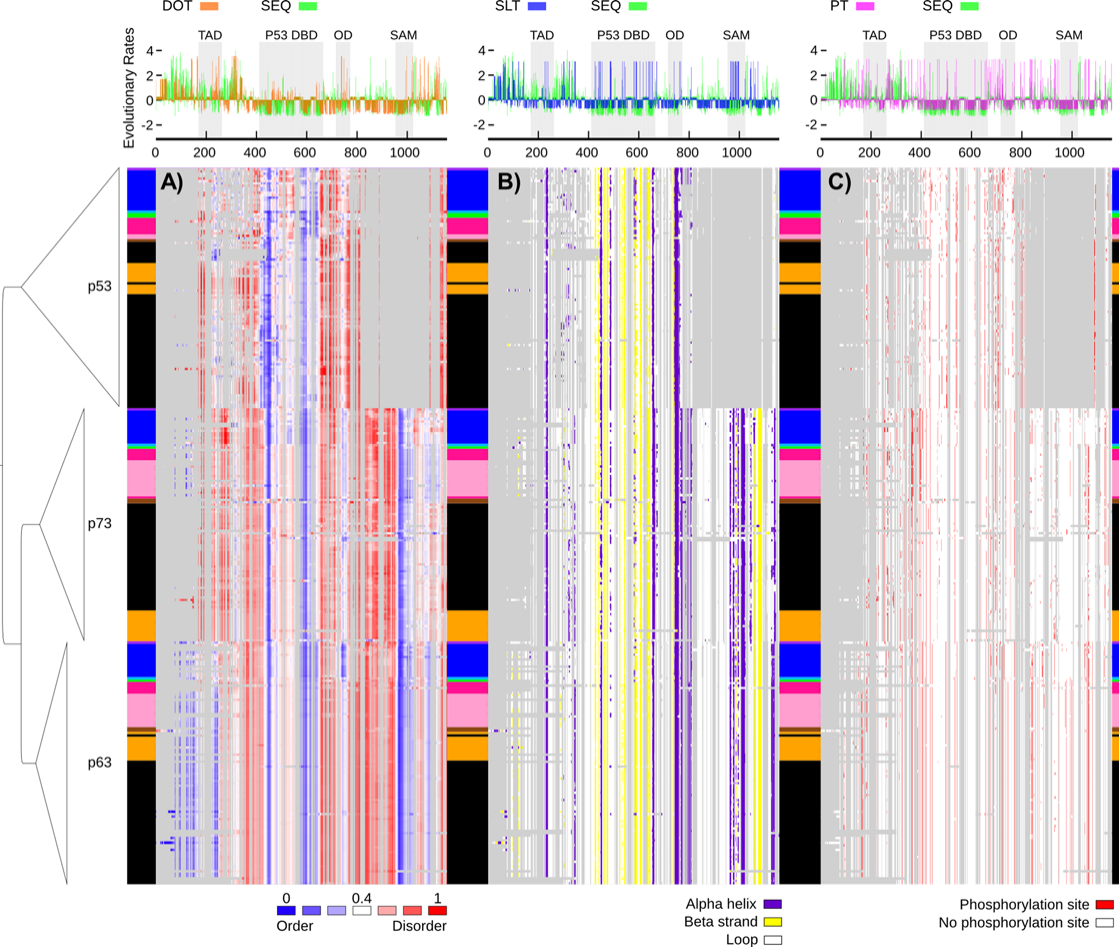
Graphical representation of sequence-based predictions in vertebrates. Heat maps for structural traits plotted in the order of the p53 DNA-based phylogenetic tree context, showing taxa names as boxes colored according to the color guide in Fig 2. The heat maps are showing sequence-based predictions mapped to their corresponding residue sites on the multiple sequence alignment (gaps in the alignment are colored in grey): (A) continuous structural disorder propensities by IUPred [15] colored according to the gradient in Fig 1, (B) secondary structure predictions by PSIPRED [24] displaying loop (white), alpha helix (purple) and beta strand (yellow), and (C) sites predicted to be phosphorylated by NetPhos [25] using a 0.75 cut-off (red). Above the heat maps, normalized evolutionary rates per site are shown for amino acid sequence (SEQ) in green [26] vs. binary traits [27] of disorder-order transitions (DOT) in orange (upper left), secondary structure elements-loop transitions (SLT) in blue (upper center), and phosphorylation transitions (PT) in pink (upper right). All evolutionary rates were normalized with a mean of zero and standard deviation of 1 (negative rates for slow evolving sites and positive rates for fast evolving sites). Grey shaded areas delimitate Pfam domain regions. For greater detail on the p53 clade, see S9 Fig.

Although the amount of structural disorder is important for the overall stability of a protein, the location of the disordered and ordered regions, as well as the multidomain context, are crucial. While p63 proteins are consistent for both disorder amount and location across species, the disorder amount and location vary greatly in p53 and p73 proteins from different species, clearly indicating that structural disorder is not conserved here (Fig 2). To address in which regions structural disorder was not conserved, the transition rate of structural disorder-order was examined across the p53 family and in the different clades. The continuous disorder propensity per residue of every protein in the p53 family was mapped onto its corresponding site in the multiple sequence alignment. The resulting heat map, with the sequences arranged corresponding to the phylogenetic tree for the p53 family, reveal interesting patterns of regions that are conserved or changing in disorder propensity (Fig 3A). To further quantify the evolutionary dynamics of structural disorder, the site specific rate of disorder-to-order transition (DOT) was inferred over the phylogeny based on a binary matrix converted from the disorder propensity heat map matrix using the same cut-off as above.

Further, amino acid (sequence) substitution rates per site (SEQ) were inferred (Fig 3). For all rates, throughout this study, positive rates evolve faster than average and negative rates evolve slower than average. DOT is faster than average in most of the p53 spanning region, except in the p53 DBD itself. For the part of the C-terminus that is missing in p53, but before the SAM domain, the sequence is diverging fast, but DOT is slow. Towards the end of SAM and in the C-terminus, p63 and p73 show rapid DOT.

### Evolutionary dynamics of secondary structure elements

With a high degree and varying amount of disorder across the p53 family, an analysis of the secondary structure elements propensities was suitable. Mapped in a heat map context, similar to that for the disorder propensity, reveal multiple regions with secondary structure transitions between sequences in the same clade and in a clade-specific manner (Fig 3). To quantify the evolutionary dynamics of secondary structure elements (alpha helix and beta strand) vs. loop across the phylogeny, a binary matrix for these properties was used to infer rates for secondary structure to loop transitions (SLT) (Fig 3). Sites with rapid SLT are found across the entire length of the alignment. Remarkably, the mostly ordered p53 DBD shows several sites with rapid SLT indicating that the structure is fluctuating among species. Also for the seemingly highly similar p63 and p73, like for the DOT, SLT is rapid in the SAM domain.

### Evolutionary dynamics of phosphorylation sites

Since phosphorylation frequently modulates the conformations of disordered regions in a regulatory fashion, an analysis of predicted phosphorylation sites was conducted. Here the heat map shows the locations of predicted phosphorylation sites in a binary fashion. Since only Ser, Thr, and Tyr can be phosphorylated, the amount of Ser, Thr, and Tyr may also be important for how many phosphorylation sites are predicted. However, while there are significant differences in the fraction of Ser, Thr, and Tyr among the different clades (p53, mean 0.17, s.d. 0.01; p63 mean 0.2, s.d. 0.01; p73, mean 0.18, s.d. 0.01) there is no significant difference in the fraction of sites predicted to be phosphorylated when comparing p53, p63 and p73 mean values (p53, mean 0.06, s.d. 0.01; p63, mean 0.06, s.d. 0.01; p73, mean 0.05, s.d. 0.01, significance based non-parametric tests with p-value < 0.05). In all clades, about 5% of all sites are predicted to be phosphorylated (S6 Fig). To quantify the evolutionary dynamics of phosphorylation sites across the phylogeny, the binary matrix was used to infer rates for presence or absence of phosphorylation sites (PT) (Fig 3). Sites with rapid PT are enriched in the linker regions.

### Functional divergence by changes in SEQ, DOT, SLT and PT rates

**Fig 4:**
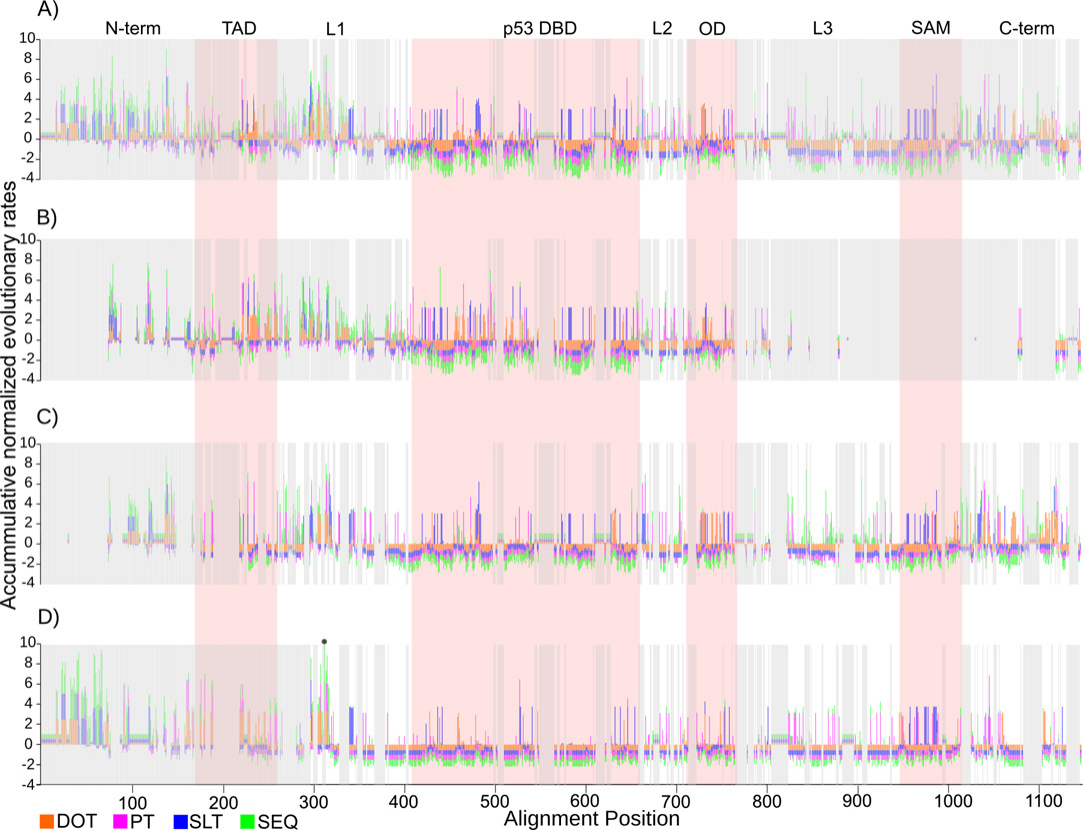
Accumulated evolutionary rates per site in vertebrates. Accumulated normalized evolutionary rates per site, (A) for the p53 family, (B-D) per clade p53, p73, and p63. SEQ, DOT, SLT, and PT colored according to Fig 2. Light pink shaded areas delimitate Pfam domain regions. Grey shaded areas have at least 10% gaps. One site with accumulated value >10 is marked with a dot.

**Fig 5:**
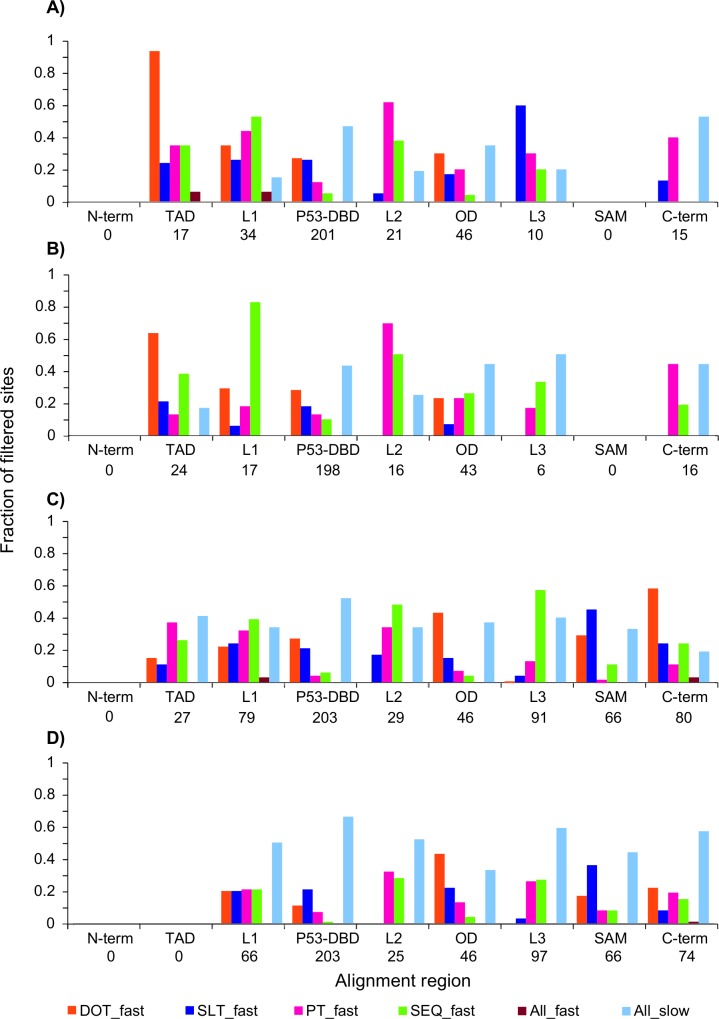
Distribution of rapid evolutionary rates per region for sites with <10% gaps in vertebrates. The number of sites with above average rates per region are shown, (A) for the p53 family, (B-D) per clade p53, p73, and p63. SEQ, DOT, SLT, and PT colored according to Fig 2. In addition, the number of sites with all rates below average (ALL_slow: light blue) and all rates above average (ALL_fast: brown) are shown. The numbers below each region label correspond to the total number of sites kept in that region after filtering out all sites with at least 10% gaps.

Regions that are rapidly changing in disorder, secondary structure, and phosphorylation are likely less important for a conserved function. These rates are calculated for the entire vertebrate p53 family and clade-specific patterns are therefore indistinct. To gain resolution on the clade level, clade-specific rates were estimated (S7 and S8 Figs). Plotting the different rates in an accumulative manner shows that gapped sites indeed have high rates (Fig 4). Since the mere presence of an indel indicates functional change, or perhaps an alternative isoform or a poorly aligned region, our attention is directed to the sites that have less than 10% gaps (Fig 4). For these sites, quantifying the number of sites with rapid DOT, SLT, PT, and SEQ, plus the number of sites that are always fast or always slow for each linker and domain region across the alignment informs which traits are diverging in the different regions (Fig 5A). Considering the p53 family level, the greater fraction of rapid DOT is found in TAD, the greater fraction of rapid SEQ is found in L1, and the greater fraction of rapid PT is found in L2. The greater fraction of rapid SLT is in L3, however, since the proteins in the p53 clade are shorter than the proteins in the p63 and p73 clades, comparisons beyond the OD domain should be made between p63 and p73 only. Considering the p53 clade (Fig 5B), TAD still has the greater fraction of rapid DOT, and L2 is still high in PT, and L1 in SEQ, but SLT is rather slow. In the p73 clade (Fig 5C), the C-terminus has the greater fraction of rapid DOT, but even the OD domain has almost half of the sites undergoing rapid DOT. SEQ is rather rapid in all linkers, and SLT is rapid for >40% of the 66 sites in the SAM domain. In the p63 clade (Fig 5D), few sites are rapid. In this clade, we note many regions with >50% of sites with all rates slow. OD from p63 and p73 have similar patterns, but more sites are rapid in SLT and PT for p63. The pattern for the OD in p53 is different. Further comparing the C-terminus of p63 to the C-terminus of p73, p63 is more constrained.

### Structural changes in regions important for molecular interactions

**Fig 6:**
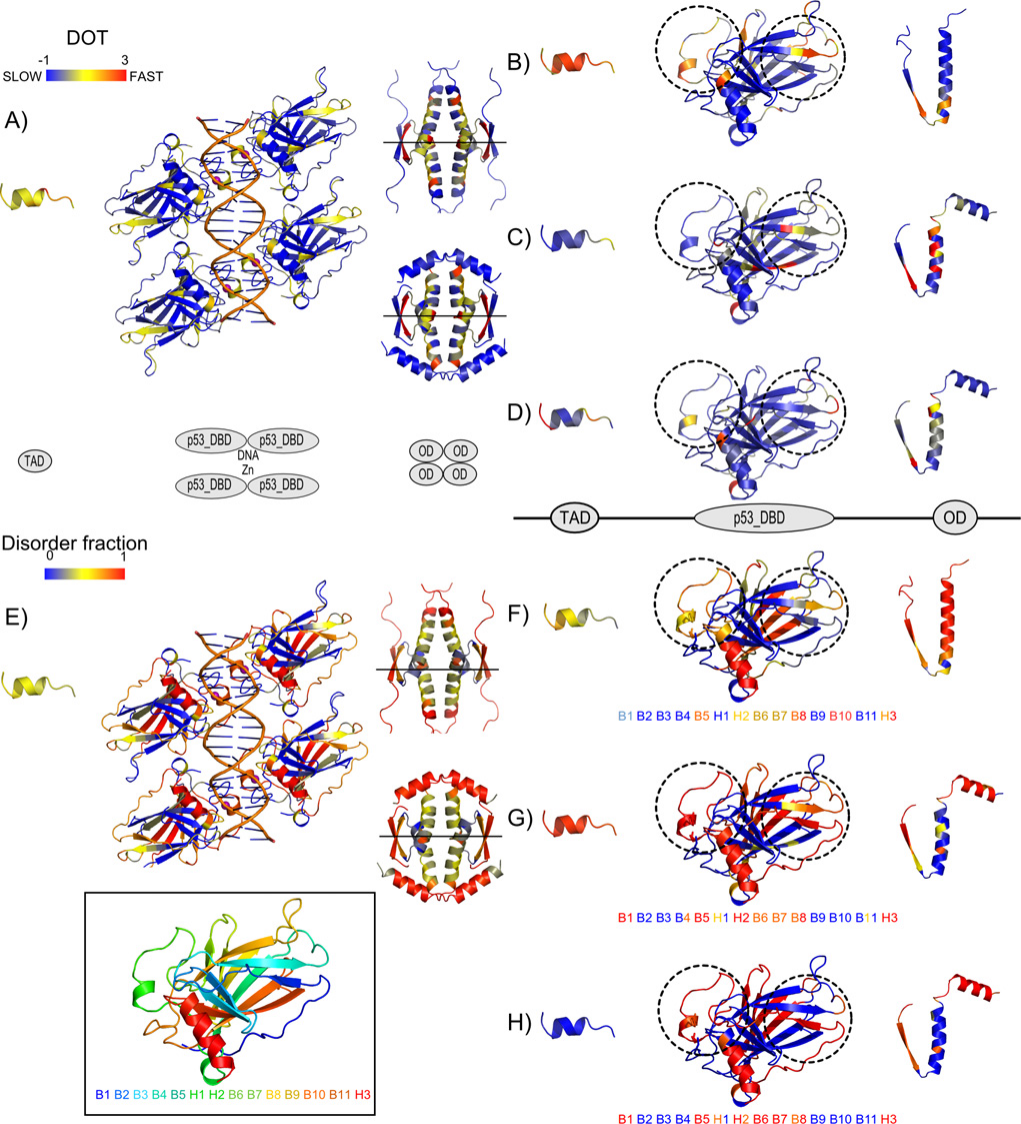
Three dimensional context of disorder-order transitions (DOT) and structural disorder conservation in vertebrates. DOT and disorder fraction (gaps included) per site are shown mapped onto representative PDB structures for TAD (PDB code 3dac [29]), p53 DBD (PDB code 4hje [30]), and OD domains (PDB code 1olg [31] for p53 and 4a9z [*To be Published*] for p63/p73); (A) DOT, and (E) disorder fraction for the p53 family showing, from left to right, TAD binding interface with MDM2, p53 DBD domains in their functional tetrameric state binding DNA and Zn as cofactor, and ODs in their functional tetrameric state (on top, values were mapped onto a p53 tetramer, and on the bottom values were mapped onto a p63 tetramer); (B-D) DOT and (F-H) disorder fraction per clade p53, p73, and p63 were mapped onto monomeric states. For further information on the ranges of the mapped regions, see S2 Table. In addition, a p53 DBD domain colored by the rainbow color scheme based on secondary structure succession (from blue to red corresponding to N-terminus and C-terminus respectively) and mapped onto a string of secondary structure elements is shown inside the box. The same string of secondary structure elements is shown in (F-H) colored by disorder fractions for an easier visualization of the differences across paralogs.

All prediction methods applied are intentionally based on linear sequences and not on 3D structures since the repertoire of 3D structures, although quite impressive for the p53 family, may only provide a limited set of snapshots of the conformational ensemble in which these proteins exist. However, the structural context is valuable and site specific DOT as well as the site specific fractions of predicted disorder were mapped onto structures for TAD, p53 DBD, and OD (all structures used were from human p53 or human p63, and only sites present in the PDB structure were mapped) (Fig 6).

For TAD, the MDM2 binding site is shown (Fig 6A and 6E). Here, moderate DOT is observed for the p53 family. On the clade level, the p53 clade shows rapid DOT (Fig 6B), p73 shows slow DOT (Fig 6C) and p63 has sites with a mixture of slow and rapid DOT (Fig 6D). For disorder conservation in TAD, on the p53 family level and on the p53 clade level intermediate conservation of disorder is observed (Fig 6E and 6F). p73 shows high conservation of disorder (Fig 6G) and p63 shows low conservation of disorder (Fig 6H).

For p53 DBD, the tetrameric state with DNA bound is displayed for the p53 family, (Fig 6A and 6E) but for each individual clade, only one of the monomers is shown (Fig 6B–6D and 6F–6H). In general, the region involved in forming the DNA binding p53 DBD dimer and in coordinating Zn as cofactor, has rapid DOT in the p53 clade, as shown in the left circle (Fig 6B). Here, p63 and p73 have slower DOT (Fig 6C and 6D) and conserved disorder (Fig 6G and 6H), while p53 has less conserved disorder (Fig 6F). The p53 clade has rapid DOT at the end of beta strand 4 (B4) and the following loop (Fig 6F, right circle). The end of the same beta strand shows rapid DOT in p73, while p63 has rapid DOT in the loop. Further, for a second beta strand (B1) in the right circle, p53 is ordered while both p63 and p73 are disordered. Lastly, one of the long beta strands (B10) in the main beta sheet has conserved disorder in p53 while p63 and p73 have conserved order.

For OD, the two different tetrameric states are displayed for the p53 family, (Fig 6A and 6E) but for each individual clade, only one of the monomers is shown (Fig 6B–6D and 6F–6H). Earlier studies of the tetramerization in p53 vs. p63 and p73 revealed that the latter two require an additional alpha helix at the C-terminus of OD in order to form stable tetramers and that heterotetramers between p63 and p73, but not p53, can form [28]. Thus, different PDB structures were used to map the functional tetrameric states for p53 and p63/p73, respectively. On the p53 family level, the area around the central horizontal axis and the ends have rapid DOT, while the rest has intermediate DOT. In the p53 clade, DOT is slow except around the horizontal axis (Fig 6B). For p63 and p73, DOT is rapid, perhaps with a slower tendency at the horizontal axis (Fig 6D and 6C). For disorder conservation in OD, p53 has conserved disorder, with slightly less conservation around the horizontal axis (Fig 6F–6H). In p73, sites are more conserved in disorder or lack of disorder, but some sites are not conserved in either property. In p63, most sites are conserved in either disorder or complete lack of disorder.

### Diverging regulation through phosphorylation

**Fig 7:**
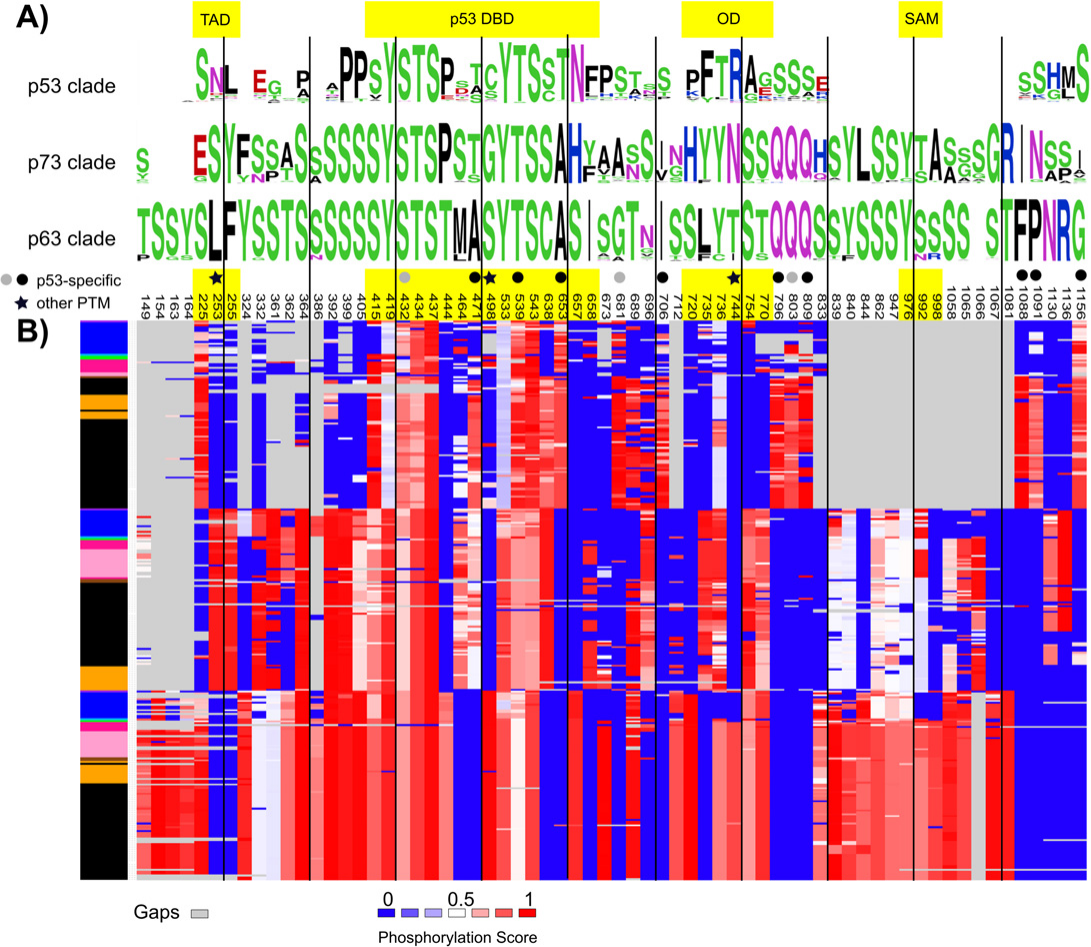
Shared and clade-specific predicted phosphorylation patterns. (A) WebLogos [35] per clade showing 66 alignment positions following a 50% majority rule of phosphorylation predictions based on a phosphorylation score cut-off = 0.75 (NetPhos), gaps included. (B) Phosphorylation predictions mapped onto their alignment sites (numeration based on the full alignment), with scores ranging from 0 (blue) to 1 (red) with 0.5 as the midpoint (white). Gaps are shown in grey. The colored boxes on the left show the distribution of species sorted by the phylogenetic tree following the color scheme as in Fig 2. Shared and clade-specific phosphorylation sites are distributed along domains (yellow shaded areas) and linkers. Sites marked with a circle means p53 clade-specific (black, the phosphorylation site is experimentally validated in PhosphoSite; grey, an adjacent site is experimentally validated to be phosphorylated in PhosphoSite). Sites marked with a star are predicted to be phosphorylated in a p63 or p73 clade-specific manner while p53 has a different experimentally verified posttranslational modification [34].

To investigate if phosphorylation may be one of the mechanisms utilized to differentiate the regulatory pathways of p53, p63, and p73 from each other, shared and clade-specific phosphorylation sites were identified using a 50% majority rule either within a clade or across the entire p53 family. In total, 66 phosphorylation sites were identified (S3 Table). Of these 66 sites, only two sites were predicted to be phosphorylated for all three clades. One, and three, sites were shared across p53/p73 and p53/p63, respectively, while eight sites were shared across p63/p73. The remaining 52 sites were clade-specific. In the p53, p63, and p73 clades, respectively, 12, 28, and 12 sites were predicted to be phosphorylated in more than 50% of the sequences for each clade. Since p53 proteins have been extensively studied, many experimental phosphorylation sites are known. For nine out of the 12 p53 clade-specific sites identified here, the NetPhos predictions are in agreement with the experimental data in the PhosphoSite database (as of Dec. 2015) that includes conserved phosphorylation sites for p53 across human, mouse, rat, rabbit and green monkey [32]. For two of the three remaining sites, the adjacent site has been experimentally validated to be phosphorylated. None of the 12 p53 clade-specific sites have been experimentally reported to be phosphorylated in PhosphoSite for either p63 or p73 homologs. For p63 and p73 clade-specific sites, no phosphorylations have been experimentally reported in PhosphoSite for the corresponding site in the p53 homologs, in agreement with the NetPhos predictions. Indeed, clade-specific positioning of phosphorylation sites in the different clades in the p53 family seem to contribute to their specific regulatory pathways. Further, not only does the phosphorylation site pattern differ between clades, but the p53 family also seem to exploit another strategy for functional diversification through shifts in the type of post-translational modification in homologous sites across paralogs. In particular, for at least three of the p63 and/or p73 clade-specific phosphorylation sites, p53 is also post-translationally modified, but with a different modification (Fig 7 and S3 Table). Alignment site 253 (TAD region) is predicted to be phosphorylated in the p73 clade (S26 in human p73). This site has Leu in most p63 sequences and Asn in some p53 sequences. For human p53, this site corresponds to Asn30 that has been found to be methylated on the carboxyl by PIMT [33,34]. Similarly, alignment site 498 (p53 DBD region) is predicted to be phosphorylated in the p63 clade (S250 in human p63). This site has Gly in all p73 sequences and Cys in some p53 sequences. For human p53, this site corresponds to Cys182 that has been found to be glutathionylated [34]. Lastly, alignment site 744 (OD region) is predicted to be phosphorylated in the p63 clade (T410 in human p63). This site has Asn in all p73 sequences and Arg in most p53 sequences. For human p53, this site corresponds to Arg337 that is known to be dimethylated [34]. Further, changes in amino acid states with compensatory effects through negatively charged amino acids were observed, e.g. alignment site 225 is phosphorylated in p53 and p63, but has Glu in p73, suggesting that p73 may resemble the phosphorylated state. Also other changes in amino acid among these sites maintain the majority of the physicochemical properties, as in Tyr-Phe transitions, while removing or adding a regulatory switch. Interestingly, some observed transitions are directly involving Ser, Thr or Tyr residues. Phosphorylation transitions between Ser/Thr (e.g. alignment site 471) are, in general, expected to conserve kinase partner and thus, conserve the regulatory mechanism, while transitions from Ser/Thr phosphorylations to Tyr phosphorylations suggest divergent mechanisms of regulation via different kinases. Alignment site 164 in p63 clade switches from Ser in ray-finned fish to Tyr in the rest of species (with shark as an exception), suggesting divergent regulation in ray-finned fish p63 proteins. Thus, differential regulation within orthologs is implied. Also, alignment site 165 is known to be phosphorylated in human p63 (Tyr36) in PhosphoSite, but this phosphorylation site is missing in all fish, where shark has Cys and the others Phe or Leu.

## Discussion

Using linear sequence predictors, properties of structural disorder (IUPred), secondary structure (PSIPRED), and phosphorylation sites (NetPhos) have been inferred. It is important to remember that these are predictions and cannot be perfect given that they are (i) independently aiming to predict traits that may depend on each other, (ii) using only the linear sequence context without considering long-range sequence contacts, and (iii) based on experimental data that may not reflect the dynamic nature of a protein sequence, e.g. one PDB structure is merely a snapshot of a conformational ensemble [36]. The accuracy for PSIPRED is >80% compared to actual experimentally determined protein structures [37]. For disordered proteins, fewer proteins are experimentally determined to be disordered. For IUPred, comparing to IDEAL (a small database of disordered proteins [number of proteins = 207]) [38] the accuracy is approximately 85%, but comparing to DisProt (a slightly larger database of disordered proteins [number of proteins = 794]) [39] the accuracy is approximately 62% [40]. However, it has been found that IUPred is more accurate in predicting order vs. disorder for DisProt proteins if the cut-off is set to 0.4 instead of the intended 0.5 [39,41]. In a different study, IUPred predictions of 0.4 were frequently found for disordered residues in partially disordered proteins [42]). Thus, we used the 0.4 cut-off to infer order vs. disorder. The sensitivity reported for NetPhos predictions cover a range from 69–96% [25], partially due to the lack of insufficient data available to train phosphorylation predictors [43]. Still, these are all standard prediction methods, widely used in computational and molecular biology when experimental data is not available.

**Fig 8:**
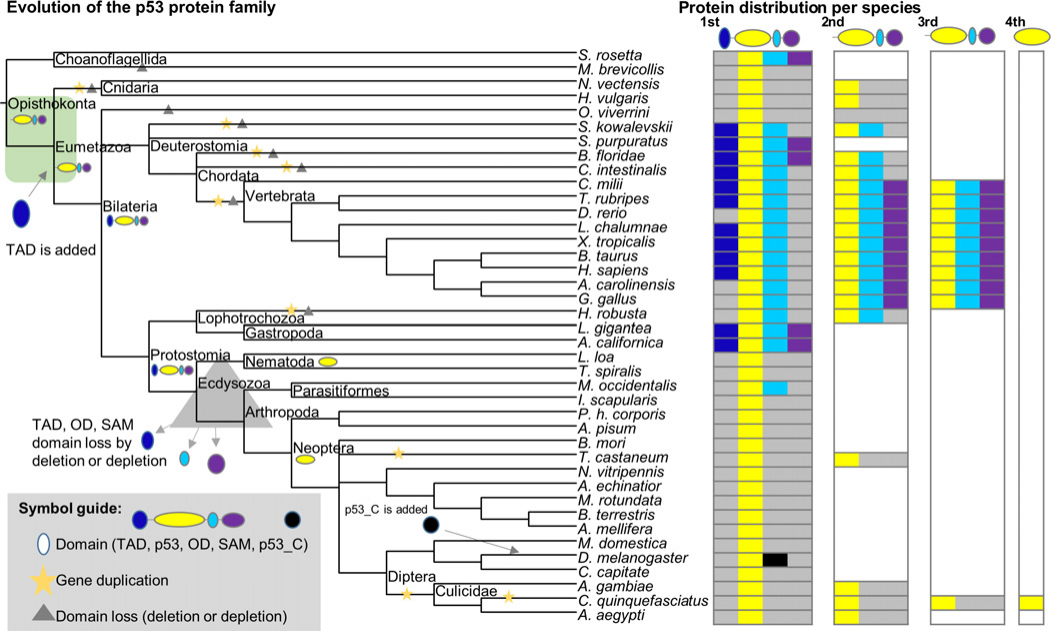
Major evolutionary events in the early p53 family. The sequences in Fig 1 are arranged by NIH Common tree taxonomy to show the evolutionary order of events (left). Branches with evidence of gene duplications are marked with a star. Branches with domain loss are marked with a triangle. Branches are not to scale. The protein distribution per species is shown (right). Presence of domains per protein are colored according to the color scheme for domains in Fig 1, with the addition that grey denotes missing domain and white denotes that no additional proteins were detected.

By comparing approximately 300 protein sequences from the vertebrate p53 family and an additional ~50 invertebrate p53 DBD domain sequences, we have investigated diverging properties from sequence to structure to regulation in the p53 family. From the invertebrate p53 DBD phylogeny, it appears that p53 DBD sequences primarily form clades based on the domain content of the full-length protein. If the p53 DBD containing proteins from Fig 1 are arranged by species in the order of taxonomy and with focus on their domain composition, a picture of the main evolutionary events of the p53 family emerges (Fig 8). As previously shown, a three domain p53 DBD containing protein is present in choanoflagellates [12]. The shared precursor of this protein and the very first metazoan p53 protein must have had at least three of the four domains found in present day vertebrate p53 family proteins. We observe proteins with all four domains in gastropods, hemichordates, and early chordates. Since these belong to Bilateria, it is clear that the bilaterian ancestor had all four domains. It should also be noted that other species not included here, such as the placozoan, *Trichoplax adhaerens*, have an MDM2 binding site [44]. Although Pfam does not classify this protein to have a TAD domain, the MDM2 binding site indicates that it does, or at least that it used to have a TAD domain. Thus, TAD predates the divergence of Bilateria and Placozoa. Further, TAD and the other non-p53 DBD domains, are frequently lost (Fig 8). In Ecdysozoa, some of these domain losses are due to actual sequence segment loss and others are due to the sequence signature being depleted. Altogether, this clearly suggests that early metazoan, and perhaps even chanoflagellates have p53 family proteins that diverged less than many of the ecdysozoan p53 family proteins that have lost most domains and frequently only consist of the p53 DBD itself. There may be other equally or more remote p53 DBD proteins in other invertebrates, like e.g. CEP-1 in *Caenorhabditis elegans* [44]. Lineage-specific gene duplications are frequent in invertebrates, but a last common ancestor of all proteins in the vertebrate p53 family is shared with *B*. *floridae* (Figs 1 and 8).

It is also clear that the p53 DBD is less structurally disordered in single domain invertebrate proteins. In vertebrates, the three paralogs p53, p63, and p73, are diverging at different rates: p63 is highly constrained while p53 is not. Ray-finned fish are demonstrating rapid lineage-specific diversification among all three paralogs. Although this study is mostly focused on the functional domains and their divergence, the inter-domain linkers vary in length and in disorder/order and secondary structure composition. Linkers are not just flexible spacers but important for controlling the conformational ensemble [45]. The divergence in linker 1 between p53 and p63 and p73 is profound and suggests functional change. TAD is rapidly diverging amongst p53 in different vertebrates, and has already diverged beyond Pfam’s domain detection ability in p63 and p73, even if some of TAD’s ancestral functionality may have remained. For p53, MDM2 is a critical regulator [44]. When MDM2 binds to key residues F19, W23, and L26 in the human p53 TAD, it can further ubiquitinate p53 on Lys residues throughout the p53 protein marking it for proteosomal degradation (reviewed in [46]). p73 was found to bind MDM2 in the same region, and although binding of MDM2 prevented p73’s transcriptional activity, it was not ubiquitinated [47]. Recently, a study found p73 to be ubiquitinated by MDM2 but p73 was not degraded [48]. For p63, the MDM2 interaction is much weaker [49]. Thus, the differential disorder among paralogs in the MDM2 binding region amongst these paralogs suggest and support divergent functional dependence on MDM2. The MDM2 binding region is frequently lost among ray-finned fish p53 proteins, and the TAD Pfam domain in general is not detected in p63 and p73, although the homologous sequence may still be there. Still, remnants of the MDM2 binding site have been found in p53 from early metazoans [44] further supporting that this is an ancestral function.

Additional indications of clade-specific functional divergence emerges from the patterns of phosphorylation. Indeed, functionally relevant phosphorylation transitions were identified and present an interesting picture of how these three paralogs have diversified in the realm of phospho-signaling. Since phosphorylation is performed by different kinases in response to various signals these seemingly small changes can allow proteins to specialize after a gene duplication. Of the three members of the p53 family, p63 is more constrained to diverge in sequence. The p63 clade has 28 clade-specific predicted phosphorylation sites above 50% conservation, compared to 12 in the p53 and p73 clades alike, suggesting that phosphorylation sites may be lost on the latter two. For at least two of the clade-specific phosphorylation sites in p63, p53 is also post-translationally modified but with a different modification, further enforcing distinct regulatory mechanisms acting on these three paralogs.

Null-mice of p63 or p73 are severely impacted and do not live long while null-mice of p53 survive to adulthood [50], suggesting that p53 is dispensable but p63 and p73 are not. The functional overlap between p53, p63, and p73 is hampered by the complexity of the protein family [51]. p53 presents lineage-specific changes and one can speculate that perhaps p53 is rapidly diversifying in a near-neutral mode due to remaining functional redundancy with p63 and p73.

p53 is a puzzling protein, known to cause and prevent cancer, prevalently mutated, in cancerous and non-cancerous cells [52]. Regardless, it cannot be expected to be functionally conserved amongst invertebrates with different domain composition, nor amongst vertebrates. Interpreting the p53 family from a molecular evolution perspective, p63 and p73 are predominantly responsible for most of the ancient function as indicated by stronger conservation of sequence and the properties here analyzed, but even in these two clades divergent regions suggest ongoing functional divergence. From a systems biology perspective, diversification in phosphorylation alters the signaling and interaction networks in which these different proteins act. From a biophysical perspective, non-conserved disorder has been interpreted as non-functional [53]. Here non-conserved disorder is found in the DNA binding region of p53, while p63 and p73 both have conserved disorder. This suggests functional diversification of the DNA binding region in p53 causing some species to become ordered in this region, perhaps bypassing a regulatory step of DNA binding regulation. Thus, an alternative interpretation for non-conserved disorder (rapid DOT) could be that it enables or disables fine-tuned signaling, rapid rewiring, or gain and loss of function(s) in a lineage-specific manner, offering a boost to biological diversity. In p53, all scenarios are possible. In the ray-finned fish clade, p53 is rapidly changing compared to the rest of the vertebrates, with many changes from fish to fish in the TAD domain. Also p63 and p73 have ray-finned fish specific changes. For p73, the p53 DBD is more ordered in ray-finned fish than in the rest of the p73 clade. For p63, the OD domain is more ordered in ray-finned fish than in the rest of the p63 clade. Co-evolution is probable. p53 from the lobe-finned fish, *L*. *chalumnae* has remarkably little disorder. Was the last common ancestor of p53 more ordered than it is today or has disorder been lost in *L*. *chalumnae*? Given that the rest of the vertebrate p53 family is more disordered, it is likely that *L*. *chalumae* has lost disorder. Without disorder, is *L*. *chalumnae*’*s* p53 still a multifunctional protein, and does it hold clues to critical, non-redundant, p53 functions, perhaps with simplified regulation? Further, what is happening to p53 in the avian genomes?

p53 is an innovative protein. While many proteins simply lose function in response to a mutation, many cancer causing mutations in p53 are thought to cause a gain-of-function [54], perhaps through mutation-driven conformational selection effects [55]. If a mutation can cause a gain-of-function, can controlled experimental conditions with wt-p53 *in vitro* have similar effects? Some gain-of-function effects seen in cancer mutants may shift the conformational ensemble since structurally disordered proteins are prone to adapt to their environmental conditions (mutation-driven conformational selection [55] vs. allosteric conformational selection [56]). Both of these effects could impact p53 *in vitro*, *in vivo*, and in a tumor cell context.

Inevitably, ongoing functional divergence is present in the p53 family, and especially in the p53 clade. The Guardian of the Genome gives the impression of still exploring its function and does not fit the picture of a resilient Guardian. Perhaps, a more appropriate way to refer to p53 is as a Gambler of the Genome?

## Methods

### Sequence retrieval

Three datasets were constructed: (i) the p53 protein family at the whole protein level in vertebrates, (ii) the p53 protein family at the nucleotide level in vertebrates, and (iii) the p53 protein family at the DNA-binding domain level in a representative set of vertebrate sequences and non-vertebrates. For (i), NCBI BLAST [57] was performed using the blastp algorithm with the human p53 protein sequence (NCBI reference sequence: NP_000537.3) against vertebrates in the RefSeq database [58]. To minimize redundancy, only the longest sequence from the same gene was chosen as the representative. Partial or much longer proteins were removed to maintain a high quality multiple sequence alignment. In some instances, sequences from key species missing in the RefSeq database were instead identified by BLAST against the nr database. For (ii), the corresponding nucleotide sequences for the amino acids sequences in (i) were retrieved from NCBI. For the final dataset (iii), NCBI BLAST was performed using the blastp algorithm with the human p53 protein DNA-binding domain excluding vertebrates in the RefSeq database to get non-vertebrate sequences. Partial proteins with an incomplete p53 DBD were removed to maintain a high quality multiple sequence alignment. To minimize redundancy and to reduce the dataset a selection of sequences was used.

For major vertebrate taxonomic groups, a representative organism with sequence information for all three paralogs in the p53 protein family was selected from (i). Vertebrate organisms included in (iii) were: *Homo sapiens*, *Bos taurus*, *Gallus gallus*, *Anolis carolinensis*, *Xenopus tropicalis*, *Latimeria chalumnae*, *Takifugu rubripes*, *Danio rerio*, and *Callorhinchus milii*. Sequence identifiers for all vertebrate sequences are given in S1 Table and protein identifiers are included in the phylogenetic trees that show sequence names.

### Phylogenetic reconstruction

Sequences for datasets (i) and (iii) were aligned with MAFFT v7.123–1 [59] using the L-INS-i algorithm for a maximum of 1000 iterations. Sequences in dataset (ii) were aligned using TranslatorX [60] to map corresponding codons to the amino acid alignment from (i). Phylogenetic trees for all datasets were constructed using MrBayes v3.2.2 [61]. For protein based phylogenies [(i) and (iii)], Bayesian MCMC analysis was performed using a mixed amino acid model with gamma distributed rate variation among sites. The nucleotide based phylogeny (ii) was estimated with Bayesian MCMC analysis using a GTR model with gamma distributed rate variation among sites. For all trees, MrBayes ran two simultaneous analyses (each with four chains: three heated and one cold) for 15 million generations with a sampling frequency of 100 generations. For dataset (i) the best tree was constructed with TBR branch swaps, while for (ii) and (iii) the best trees were constructed with TBR branch swaps disabled. The final average standard deviation of the split frequencies were 0.0060 (max. s.d. 0.051) for dataset (i), 0.0053 (max. s.d. 0.092) for dataset (ii), and 0.0023 (max. s.d. 0.016) for dataset (iii). Consensus trees were built with the default burn-in phase (discarding the first 25% of trees) using the 50% majority rule. The tree from the third dataset was rooted on a branch containing *Monosiga brevicollis* and *Salpingoeca rosetta*. The resulting topology was used to guide rooting the trees from the first two datasets by rooting on the branch containing both p63 and p73 clades and selecting the p53 clade as the outgroup.

### Sequence-based predictions

To assess the characterization of the structural properties of the proteins included in our phylogenies, the amino acid sequence of each protein (unaligned sequence) was used as input for different sequence-based predictors in order to predict structural disorder, secondary structure, phosphorylation sites and domain regions. Thereafter, for each prediction method, the predicted value for each residue in each protein sequence was mapped onto its corresponding site in the multiple sequence alignment. This resulted in three matrices for (i) structural disorder prediction, (ii) secondary structure predictions, and (iii) predicted phosphorylation sites. For (i) and (iii), the data predicted was continuous. For (ii), the data had three non-numerical categories. In order to analyze the transitions between order and disorder, between the presence of secondary structure elements and loops, and for presence or absence of phosphorylation sites, all matrices were represented as binary phyletic patterns (as described below). The phyletic patterns were individually analyzed in their phylogenetic context and transition rates were calculated.

### Structural disorder prediction

Structural disorder was predicted using IUPred [15,62] version 1.0 selecting the option for long disordered regions. IUPred was specifically developed for predicting disorder in intrinsically unfolded proteins using estimated energy content. The IUPred prediction generates a disorder propensity for each residue in the protein. The disorder propensities range from 0 (indicating no propensity of being disordered) to 1 (indicating strong propensity of being disordered). While the method was developed to have scores above 0.5 indicating disorder, a cut-off of 0.4 was later demonstrated to give higher accuracy when predicting disorder on proteins from the experimentally verified DisProt database [41,42]. The continuous disorder predictions were mapped onto the multiple sequence alignment, and visualized in a heat map format using iTOL [63]. Further, all sites with IUPred prediction values <0.4 were assigned order and all sites ≥0.4 were assigned disorder. This binary matrix was used as a phyletic pattern for analyzing the evolutionary dynamics of structural disorder to order transitions (DOT).

### Secondary structure prediction

Secondary structure was predicted using PSIPRED [24,64] version 3.4 with default parameters and the nr database (version March.30.2014), filtered to avoid low complexity regions, coiled-coil regions and transmembrane regions, was selected to generate a sequence profile per protein. PSIPRED is a neural network program which performs an analysis on the sequence profiles obtained from PsiBlast (Position Specific Iterated–BLAST version 2.2.26, blastpgp) [65] converting them to secondary structure propensities. The three states of secondary structure propensity (alpha helix, beta strand, and loop) were visualized in a heat map. The PSIPRED predictions were converted into binary data: alpha helix/beta strand residues were set to 1 and loop residues were set to 0. This binary matrix was used as a phyletic pattern for analyzing the evolutionary dynamics of secondary structure to loop transitions (SLT).

### Phosphorylation site prediction

Phosphorylation sites for Serine, Threonine and Tyrosine residues were predicted using NetPhos [25] version 3.1, an artificial neural network method. Similar to the other predictions, two states were defined: sites with values <0.75 were assigned not phosphorylated or 0 and all sites ≥0.75 were assigned as sites predicted to be phosphorylated or 1. Sites predicted to be phosphorylated were visualized in a heat map. The resulting binary matrix was used for analyzing the evolutionary dynamics of phosphorylation transitions (PT).

### Protein domain prediction

Protein domains were predicted based on Pfam [66] version 27 by aligning each sequence to their stored Hidden Markov Model (HMM) profiles using the available batch search scripts. Sites in domains with significant bit scores based on pre-defined gathering thresholds, predicted to be part of a Pfam_A domain (based on the envelope coordinates), were visualized in a heat map.

### Evolutionary dynamics of sequence data

Rate4Site [67] was used to estimate the amino acid substitution rates (SEQ) by an empirical Bayesian principle under the Jones, Taylor, and Thornton [68] amino acids substitution model (JTT) using a prior gamma distribution including 16 discrete categories. Rate4Site estimates the site specific rates considering the topology and branch lengths of the phylogenetic tree. The branch lengths were not optimized as the input trees were obtained by Bayesian inference. Normalized evolutionary rates in Rate4Site are Z-scores, scaled such that the average across all sites is equal to zero and standard deviation is equal to 1. This means that sites showing a normalized evolutionary rate <0 are evolving slower than average, and those with a rate >0 are evolving faster than average.

### Evolutionary dynamics of predicted data

To study the gain/loss transitions of structural properties in related proteins along their evolutionary history, a protocol that includes the estimation of evolutionary rates per site based on the phylogenetic trees and the binary matrices generated was adopted. GLOOME software [27] was used to study the evolutionary dynamics of structural disorder (DOT rate; disorder-order transitions), secondary structures (SLT rate; secondary structure-loop transitions), and phosphorylation sites (PT rate; phosphorylation transitions). GLOOME was originally developed to study the gain/loss events across phylogenies. Here GLOOME was applied to analyze trends in binary presence (1) and absence (0) patterns in predicted protein sequence features (disorder vs. no-disorder, secondary structure vs. no secondary structure, phosphorylation site vs. no phosphorylation site) with default equal substitution rates for transitions within the same state (0 to 0, 1 to 1) and default equal rates for substitutions form one state to another (0 to 1, 1 to 0) and a rate distribution of 6 gamma categories. The outputs include the evolutionary rates per alignment site normalized as a Z-score (the same way as for the sequence data in Rate4Site). Lastly, for each of the evolutionary rates calculated (SEQ, DOT, SLT and PT) for the family and the individual clades, we further analyzed those aligned sites with less than 10% of gaps per alignment position.

### Non-parametric tests

Inference methods implemented in R statistical software [69] were used for testing if differences in means across groups are statistically significant (p-value <0.05). According to the Shapiro-Wilk test [70], normality could not be assumed and non-parametric tests were performed. For three or more samples the Kruskal-Wallis test [71] was applied, while the pairwise testing involved the use of the Mann-Whitney U test with Bonferroni correction [72,73].

### 3D mapping of structural disorder conservation and disorder-to-order transition rates

Conservation, here defined as the fraction of disorder per site from the binary matrices (gaps included), was calculated for the p53 family and the individual clades. Site specific rates and conservation of disorder were mapped onto representative PDB structures for the different domains. S2 Table shows the details of the mapped regions. Figures were generated using PYMOL [74].

## Supporting Information

S1 Fig p53 domain phylogeny for Metazoa and Choanoflagellates. Overview of the p53 family phylogeny including 74 representative species across Metazoa and Choanoflagellates, built based on their p53 DBD domains. Support values at the nodes indicate posterior probabilities. Nodes with posterior probability < 0.5 are unresolved.
(PDF)

S2 Fig p53 Phylogenies for 301 Vertebrate Proteins. (A) Circular representations of p53 DNA-based phylogeny and (B) its corresponding full-protein-based phylogeny. These consensus trees were obtained with MrBayes 3.2.2 after sampling trees for 15 million generations with the default burn-in phase (discarding the first 25% of trees) and using the 50% majority rule. Node circles show posterior probabilities ranging from 0.5 in red to 1 in white. Here proteins were colored by clade (p53 in grey, p63 in blue and p73 in green) with tip labels following the color guide from Fig 2. Figure generated with FigTree (http://tree.bio.ed.ac.uk/software/figtree/).
(PDF)

S3 Fig Domain Composition in Vertebrate Proteins. (A) Heat map showing Pfam domain predictions per protein into their corresponding multiple sequence alignment sites (rows show protein hits; columns show alignment positions; sites that belong to Pfam_A domains are colored, green; linkers between domains, white; gaps in the alignment, grey), all in the context of the p53 DNA-based phylogeny with tip labels colored according to the color guide. (B) In addition, individual domain architectures (labeled and colored as shown in Pfam domains box) were also included to highlight their actual lengths enforcing missing or broken domains. Figure generated with iTOL [63].
(PDF)

S4 Fig Structural Disorder Fractions in Vertebrate Proteins. Distribution of structural disorder (grey) and order (blue) in full-length proteins and in p53 DBD domains sorted by p53 DNA-based phylogenetic tree with tip labels following the color guide. Furthermore, individual domain architectures (labeled and colored as shown in Pfam domains box) were also included. Figure generated with iTOL [63].
(PDF)

S5 Fig p53 DBD Structural Disorder Content Increases with the Number of Domains. Scatter plot of the p53 DBD structural disorder percentage vs. the number of Pfam domains per protein from 74 hits, including invertebrates and vertebrates proteins. There is a positive correlation between these two variables (Pearson correlation coefficient R = 0.64, R^2^ = 0.41, and p-value < 0.05, concluding that linear correlation different to 0 is statistically significant).
(PDF)

S6 Fig Differential Distribution of Protein Phosphorylations per Clade. Boxplots showing the fractions of serine, threonine and tyrosine residues per protein per clade compared to the fractions of sites predicted to be phosphorylated by NetPhos [25] using a 0.75 cut-off. Significance analysis was carried out using non-parametric tests (Kruskal Wallis test for the comparison of 3 or more samples and Mann-Whitney U test with Bonferroni correction for the pairwise analysis). Differences in means are statistically significant (p-values << 0.05), except for the p53-p63 comparison of predicted phosphorylation factions (p-value = 1).
(PDF)

S7 Fig Comparison of SEQ, SLT and PT with DOT rates. Combined profiles of normalized evolutionary rates per aligned site for family and clades (vertebrates set) comparing disorder-order transitions (DOT) with (A) amino acid substitutions (SEQ), (B) secondary structure elements-loop transitions (SLT), and (C) phosphorylation transitions (PT). Grey shaded areas delimitate Pfam domain regions.
(PDF)

S8 Fig Comparison of SLT and PT with SEQ rates. Combined profiles of normalized evolutionary rates per aligned site for family and clades (vertebrates set) comparing amino acid substitutions (SEQ) with (A) secondary structure elements-loop transitions (SLT) and (B) phosphorylation transitions (PT). Grey shaded areas delimitate Pfam [66] domain regions.
(PDF)

S9 Fig p53 clade in detail: graphical representation of sequence-based predictions. Heat map for structural traits plotted in the order of the p53 DNA-based phylogenetic tree context, showing p53 protein names as boxes colored according to the color guide in Fig 2. These heat maps are showing sequence-based predictions mapped to their corresponding residue sites in the multiple sequence alignment, after removing empty columns (i.e. columns fully gapped in the p53 clade) for this subset: (A) continuous structural disorder propensities by IUPred [15,62] with a color gradient from blue to white to red mirroring the disorder propensity gradient from low (blue) to high (red), with white being the boundary between order and disorder (remaining alignment gaps are colored in grey). (B) secondary structure predictions by PSIPRED [24,64] displaying 3 states loop (white), alpha helix (purple) and beta strand (yellow), and C) sites predicted to be phosphorylated by NetPhos [25] using a 0.75 cut-off (red). On top of these heat maps, normalized evolutionary rates per site are shown for amino acid sequence (SEQ) in green [26] vs. binary traits [27] of disorder-order transitions (DOT) in orange (upper left), secondary structure elements—loop transitions (SLT) in blue (upper center), and phosphorylation transitions (PT) in pink (upper right). All evolutionary rates were normalized with a mean of zero and standard deviation of 1: negative rates for slow evolving sites and positive rates for fast evolving sites. Grey shaded areas delimitate Pfam domain regions.
(PDF)

S1 Table Accession numbers for the vertebrate datasets (i) and (ii).
(PDF)

S2 Table PDB files and regions used for mapping DOT and disorder conservation into a structural context. (PDF)

S3 Table Shared and clade specific predicted phosphorylation patterns. Alignment sites following a 50% majority rule of sequences with phosphorylation predictions based on NetPhos phosphorylation prediction score cut-off = 0.75 (gaps included). Information displayed per clade (specific) and per family (shared). Shaded areas correspond to the majority rule (phosphorylation predicted for more than 50% of taxa per clade or for the family). Corresponding positions in the canonical human proteins (P53_human NP_000537.3, P63_human NP_003713.3, and P73_human NP_005418.1) are shown.
(PDF)

## References

[1] Gunasekaran K, Ma B, Nussinov R (2004) Is allostery an intrinsic property of all dynamic proteins? Proteins 57: 433–443. 15382234 10.1002/prot.20232

[2] Tokuriki N, Tawfik DS (2009) Protein dynamism and evolvability. Science 324: 203–207. 10.1126/science.1169375 19359577

[3] Siltberg-Liberles J (2011) Evolution of structurally disordered proteins promotes neostructuralization. Mol Biol Evol 28: 59–62. 10.1093/molbev/msq291 21037204 PMC3108607

[4] Vogelstein B, Lane D, Levine AJ (2000) Surfing the p53 network. Nature 408: 307–310. 11099028 10.1038/35042675

[5] Collavin L, Lunardi A, Del Sal G (2010) p53-family proteins and their regulators: hubs and spokes in tumor suppression. Cell Death Differ 17: 901–911. 10.1038/cdd.2010.35 20379196

[6] Uversky VN, Oldfield CJ, Dunker AK (2008) Intrinsically disordered proteins in human diseases: Introducing the D(2) concept. 37: 215–246. 10.1146/annurev.biophys.37.032807.125924 18573080

[7] Oldfield CJ, Meng J, Yang JY, Yang MQ, Uversky VN, Dunker AK (2008) Flexible nets: disorder and induced fit in the associations of p53 and 14-3-3 with their partners. BMC Genomics 9 Suppl 1: S1 10.1186/1471-2164-9-S1-S1 18366598 PMC2386051

[8] Yu Q, Ye W, Wang W, Chen H-F (2013) Global conformational selection and local induced fit for the recognition between intrinsic disordered p53 and CBP. PLoS One 8: e59627 10.1371/journal.pone.0059627 23555731 PMC3608666

[9] Xue B, Brown CJ, Dunker AK, Uversky VN (2013) Intrinsically disordered regions of p53 family are highly diversified in evolution. Biochim Biophys Acta 1834: 725–738. 10.1016/j.bbapap.2013.01.012 23352836 PMC3905691

[10] Wagner A (2008) Neutralism and selectionism: a network-based reconciliation. Nat Rev Genet 9: 965–974. 10.1038/nrg2473 18957969

[11] Assis R, Kondrashov AS (2014) Conserved proteins are fragile. Mol Biol Evol 31: 419–424. 10.1093/molbev/mst217 24202613 PMC3907047

[12] Nedelcu AM, Tan C (2007) Early diversification and complex evolutionary history of the p53 tumor suppressor gene family. Dev Genes Evol 217: 801–806. 17924139 10.1007/s00427-007-0185-9

[13] Putnam NH, Butts T, Ferrier DEK, Furlong RF, Hellsten U, Kawashima T, et al (2008) The amphioxus genome and the evolution of the chordate karyotype. Nature 453: 1064–1071. 10.1038/nature06967 18563158

[14] Berná L, Alvarez-Valin F (2014) Evolutionary genomics of fast evolving tunicates. Genome Biol Evol 6: 1724–1738. 10.1093/gbe/evu122 25008364 PMC4122922

[15] Dosztányi Z, Csizmok V, Tompa P, Simon I (2005) IUPred: web server for the prediction of intrinsically unstructured regions of proteins based on estimated energy content. Bioinformatics 21: 3433–3434. 15955779 10.1093/bioinformatics/bti541

[16] Lane DP, Madhumalar A, Lee AP, Tay B-H, Verma C, Brenner S, et al (2011) Conservation of all three p53 family members and Mdm2 and Mdm4 in the cartilaginous fish. Cell Cycle 10: 4272–4279. 10.4161/cc.10.24.18567 22107961 PMC3272259

[17] Soussi T, Bègue A, Kress M, Stehelin D, May P (1988) Nucleotide sequence of a cDNA encoding the chicken p53 nuclear oncoprotein. Nucleic Acids Res 16: 11383 3060861 10.1093/nar/16.23.11383 PMC339033

[18] International Chicken Genome Sequencing Consortium. Sequence and comparative analysis of the chicken genome provide unique perspectives on vertebrate evolution. (2004). Nature 432: 695–716. 15592404 10.1038/nature03154

[19] Belyi VA, Ak P, Markert E, Wang H, Hu W, Puzio-Kuter A, et al (2010) The origins and evolution of the p53 family of genes. Cold Spring Harb Perspect Biol 2: a001198 10.1101/cshperspect.a001198 20516129 PMC2869528

[20] Cai Q, Qian X, Lang Y, Luo Y, Xu J, Pan S, et al (2013) Genome sequence of ground tit Pseudopodoces humilis and its adaptation to high altitude. Genome Biol 14: R29 10.1186/gb-2013-14-3-r29 23537097 PMC4053790

[21] Bornberg-Bauer E, Beaussart F, Kummerfeld SK, Teichmann SA, Weiner J (2005) The evolution of domain arrangements in proteins and interaction networks. Cell Mol Life Sci 62: 435–445. 15719170 10.1007/s00018-004-4416-1 PMC11924419

[22] Venkatesh B, Lee AP, Ravi V, Maurya AK, Lian MM, Swann JB, et al (2014) Elephant shark genome provides unique insights into gnathostome evolution. Nature 505: 174–179. 10.1038/nature12826 24402279 PMC3964593

[23] Brandt T, Kaar JL, Fersht AR, Veprintsev DB (2012) Stability of p53 homologs. PLoS One 7: e47889 10.1371/journal.pone.0047889 23112865 PMC3480436

[24] McGuffin LJ, Bryson K, Jones DT (2000) The PSIPRED protein structure prediction server. Bioinformatics 16: 404–405. 10869041 10.1093/bioinformatics/16.4.404

[25] Blom N, Gammeltoft S, Brunak S (1999) Sequence and structure-based prediction of eukaryotic protein phosphorylation sites. J Mol Biol 294: 1351–1362. 10600390 10.1006/jmbi.1999.3310

[26] Pupko T, Bell RE, Mayrose I, Glaser F, Ben-Tal N (2002) Rate4Site: an algorithmic tool for the identification of functional regions in proteins by surface mapping of evolutionary determinants within their homologues. Bioinformatics 18: S71–S77. 12169533 10.1093/bioinformatics/18.suppl_1.s71

[27] Cohen O, Ashkenazy H, Belinky F, Huchon D, Pupko T (2010) GLOOME: gain loss mapping engine. Bioinformatics 26: 2914–2915. 10.1093/bioinformatics/btq549 20876605

[28] Joerger AC, Wilcken R, Andreeva A (2014) Tracing the evolution of the p53 tetramerization domain. Structure 22: 1301–1310. 10.1016/j.str.2014.07.010 25185827 PMC4155161

[29] Popowicz GM, Czarna A, Holak TA (2008) Structure of the human Mdmx protein bound to the p53 tumor suppressor transactivation domain. Cell Cycle 7: 2441–2443. 18677113 10.4161/cc.6365

[30] Chen Y, Zhang X, Dantas Machado AC, Ding Y, Chen Z, Qin PZ, et al (2013) Structure of p53 binding to the BAX response element reveals DNA unwinding and compression to accommodate base-pair insertion. Nucleic Acids Res 41: 8368–8376. 10.1093/nar/gkt584 23836939 PMC3783167

[31] Clore GM, Omichinski JG, Sakaguchi K, Zambrano N, Sakamoto H, Apella E, et al (1994) High-resolution structure of the oligomerization domain of p53 by multidimensional NMR. Science 265: 386–391. 8023159 10.1126/science.8023159

[32] Hornbeck P V, Zhang B, Murray B, Kornhauser JM, Latham V, Skrzypek E (2015) PhosphoSitePlus, 2014: mutations, PTMs and recalibrations. Nucleic Acids Res 43: D512–D520. 10.1093/nar/gku1267 25514926 PMC4383998

[33] Lee J-C, Kang S-U, Jeon Y, Park JW, You J-S, Ha SW, et al (2012) Protein L-isoaspartyl methyltransferase regulates p53 activity. Nat Commun 3: 927 10.1038/ncomms1933 22735455 PMC3621463

[34] Nguyen T-A, Menendez D, Resnick MA, Anderson CW (2014) Mutant TP53 posttranslational modifications: challenges and opportunities. Hum Mutat 35: 738–755. 10.1002/humu.22506 24395704 PMC4074372

[35] Crooks GE, Hon G, Chandonia J-M, Brenner SE (2004) WebLogo: a sequence logo generator. Genome Res 14: 1188–1190. 15173120 10.1101/gr.849004 PMC419797

[36] Slabinski L, Jaroszewski L, Rodrigues APC, Rychlewski L, Wilson IA, Lesley SA, et al (2007) The challenge of protein structure determination—lessons from structural genomics. Protein Sci 16: 2472–2482. 17962404 10.1110/ps.073037907 PMC2211687

[37] Bryson K, McGuffin LJ, Marsden RL, Ward JJ, Sodhi JS, Jones DT (2005) Protein structure prediction servers at University College London. Nucleic Acids Res 33: W36–W38. 15980489 10.1093/nar/gki410 PMC1160171

[38] Fukuchi S, Sakamoto S, Nobe Y, Murakami SD, Amemiya T, Hosoda K (2012) IDEAL: Intrinsically Disordered proteins with Extensive Annotations and Literature. Nucleic Acids Res 40: D507–D511. 10.1093/nar/gkr884 22067451 PMC3245138

[39] Sickmeier M, Hamilton JA, LeGall T, Vacic V, Cortese MS, Tantos A, et al (2007) DisProt: the Database of Disordered Proteins. Nucleic Acids Res 35: D786–D793. 17145717 10.1093/nar/gkl893 PMC1751543

[40] Di Domenico T, Walsh I, Tosatto SCE (2013) Analysis and consensus of currently available intrinsic protein disorder annotation sources in the MobiDB database. BMC Bioinformatics 14 Suppl 7: S3 10.1186/1471-2105-14-S7-S3 23815411 PMC3633070

[41] Fuxreiter M, Tompa P, Simon I (2007) Local structural disorder imparts plasticity on linear motifs. Bioinformatics 23: 950–956. 17387114 10.1093/bioinformatics/btm035

[42] Xue B, Oldfield CJ, Dunker AK, Uversky VN (2009) CDF it all: consensus prediction of intrinsically disordered proteins based on various cumulative distribution functions. FEBS Lett 583: 1469–1474. 10.1016/j.febslet.2009.03.070 19351533 PMC2683465

[43] Trost B, Kusalik A (2011) Computational prediction of eukaryotic phosphorylation sites. Bioinformatics 27: 2927–2935. 10.1093/bioinformatics/btr525 21926126

[44] Lane DP, Cheok CF, Brown C, Madhumalar A, Ghadessy FJ, Verma C (2010) Mdm2 and p53 are highly conserved from placozoans to man. Cell Cycle. 10.4161/cc.9.3.10516 20081368

[45] Ma B, Tsai C-J, Haliloğlu T, Nussinov R (2011) Dynamic allostery: linkers are not merely flexible. Structure 19: 907–917. 10.1016/j.str.2011.06.002 21742258 PMC6361528

[46] Chao CC-K (2015) Mechanisms of p53 degradation. Clin Chim Acta 438: 139–147. 10.1016/j.cca.2014.08.015 25172038

[47] Bálint E, Bates S, Vousden KH (1999) Mdm2 binds p73 alpha without targeting degradation. Oncogene 18: 3923–3929. 10435614 10.1038/sj.onc.1202781

[48] Wu H, Leng RP (2015) MDM2 mediates p73 ubiquitination: a new molecular mechanism for suppression of p73 function. Oncotarget 6: 21479–21492. 26025930 10.18632/oncotarget.4086 PMC4673280

[49] Zdzalik M, Pustelny K, Kedracka-Krok S, Huben K, Pecak A, Wladyka B, et al (2014) Interaction of regulators Mdm2 and Mdmx with transcription factors p53, p63 and p73. Cell Cycle 9: 4584–4591. 10.4161/cc.9.22.13871 21088494

[50] Stiewe T (2007) The p53 family in differentiation and tumorigenesis. Nat Rev Cancer 7: 165–168. 17332760 10.1038/nrc2072

[51] Costanzo A, Pediconi N, Narcisi A, Guerrieri F, Belloni L, Fausti F, et al (2014) TP63 and TP73 in cancer, an unresolved “family” puzzle of complexity, redundancy and hierarchy. FEBS Lett 588: 2590–2599. 10.1016/j.febslet.2014.06.047 24983500

[52] Martincorena I, Roshan A, Gerstung M, Ellis P, Van Loo P, McLaren S, et al (2015) High burden and pervasive positive selection of somatic mutations in normal human skin. Science (80-) 348: 880–886. 10.1126/science.aaa6806 PMC4471149 25999502

[53] van der Lee R, Buljan M, Lang B, Weatheritt RJ, Daughdrill GW, Dunker KA, et al (2014) Classification of intrinsically disordered regions and proteins. Chem Rev 114: 6589–6631. 10.1021/cr400525m 24773235 PMC4095912

[54] Brosh R, Rotter V (2009) When mutants gain new powers: news from the mutant p53 field. Nat Rev Cancer 9: 701–713. 10.1038/nrc2693 19693097

[55] Siltberg-Liberles J, Grahnen JA, Liberles DA (2011) The Evolution of Protein Structures and Structural Ensembles Under Functional Constraint. Genes (Basel) 2: 748–762. 10.3390/genes2040748 24710290 PMC3927589

[56] Nussinov R, Ma B, Tsai C-J (2014) Multiple conformational selection and induced fit events take place in allosteric propagation. Biophys Chem 186: 22–30. 10.1016/j.bpc.2013.10.002 24239303 PMC6361548

[57] Altschul SF, Gish W, Miller W, Myers EW, Lipman DJ (1990) Basic local alignment search tool. J Mol Biol 245: 403–410. 10.1016/S0022-2836(05)80360-2 2231712

[58] Pruitt KD, Tatusova T, Maglott DR (2005) NCBI Reference Sequence (RefSeq): a curated non-redundant sequence database of genomes, transcripts and proteins. Nucleic Acids Res 33: D501–D504. 10.1093/nar/gki025 15608248 PMC539979

[59] Katoh K, Misawa K, Kuma K, Miyata T (2002) MAFFT: a novel method for rapid multiple sequence alignment based on fast Fourier transform. Nucleic Acids Res 30: 3059–3066. 12136088 10.1093/nar/gkf436 PMC135756

[60] Abascal F, Zardoya R, Telford MJ (2010) TranslatorX: multiple alignment of nucleotide sequences guided by amino acid translations. Nucleic Acids Res 38: W7–W13. 10.1093/nar/gkq291 20435676 PMC2896173

[61] Ronquist F, Teslenko M, van der Mark P, Ayres DL, Darling A, Höhna S, et al (2012) MrBayes 3.2: Efficient Bayesian Phylogenetic Inference and Model Choice Across a Large Model Space. Softw Syst Evol 61: 539–542. 10.1093/sysbio/sys029 PMC3329765 22357727

[62] Dosztányi Z, Csizmók V, Tompa P, Simon I (2005) The pairwise energy content estimated from amino acid composition discriminates between folded and intrinsically unstructured proteins. J Mol Biol 347: 827–839. 10.1016/j.jmb.2005.01.071 15769473

[63] Letunic I, Bork P (2007) Interactive Tree Of Life (iTOL): an online tool for phylogenetic tree display and annotation. Bioinformatics 23: 127–128. 10.1093/bioinformatics/btl529 17050570

[64] Jones DT (1999) Protein secondary structure prediction based on position-specific scoring matrices. J Mol Biol 292: 195–202. 10.1006/jmbi.1999.3091 10493868

[65] Altschul SF, Madden TL, Schäffer AA, Zhang J, Zhang Z, Miller W, et al (1997) Gapped BLAST and PSI-BLAST: a new generation of protein database search programs. Nucleic Acids Res 25: 3389–3402. 9254694 10.1093/nar/25.17.3389 PMC146917

[66] Finn RD, Bateman A, Clements J, Coggill P, Eberhardt RY, Eddy SR, et al (2014) Pfam: the protein families database. Nucleic Acids Res 42: D222–D230. 10.1093/nar/gkt1223 24288371 PMC3965110

[67] Mayrose I, Graur D, Ben-Tal N, Pupko T (2004) Comparison of site-specific rate-inference methods for protein sequences: empirical Bayesian methods are superior. Mol Biol Evol 21: 1781–1791. 15201400 10.1093/molbev/msh194

[68] Jones DT, Taylor WR, Thornton JM (1992) The rapid generation of mutation data matrices from protein sequences. Comput Appl Biosci 8: 275–282. 1633570 10.1093/bioinformatics/8.3.275

[69] The R Core Team (2012) R: A language and environment for statistical computing R Foundation for Statistical Computing, Vienna, Austria.

[70] Shapiro SS, Wilk MB (1965) An Analysis of Variance Test for Normality (Complete Samples). Biometrika 52: 591–611.

[71] Kruskal WH, Wallis WA (1952) Use of Ranks in One-Criterion Variance Analysis. J Am Stat Assoc 47: 583–621.

[72] Mann HB, Whitney DR (1947) On a Test of Whether one of Two Random Variables is Stochastically Larger than the Other. Ann Math Stat 18: 50–60.

[73] Dunn OJ (1961) Multiple Comparisons among Means. J Am Stat Assoc 56: 52–64.

[74] DeLano WL (n.d.) The PyMOL Molecular Graphics System, Schrödinger, LLC. Available: https://sourceforge.net/projects/pymol/

