# Supplementary material for: Functional Diversification after Gene Duplication: Paralog Specific Regions of Structural Disorder and Phosphorylation in p53, p63, and p73
Source: PLoS One. 2016 Mar 22;11(3):e0151961. doi: 10.1371/journal.pone.0151961 (PMC4803236; doi:10.1371/journal.pone.0151961)
Supplement: S1 Fig — Overview of the p53 family phylogeny including 74 representative species across Metazoa and Choanoflagellates, built based on their p53 DBD domains. Support values at the nodes indicate posterior probabilities. Nodes with posterior probability < 0.5 are unresolved. (PDF) [file pone.0151961.s001.pdf]

Supplementary material

S1 Fig. p53 domain phylogeny for Metazoa and Choanoflagellates. Overview of the p53 family phylogeny including 74 representative species across Metazoa and Choanoflagellates, built based on their p53 DBD domains. Support values at the nodes indicate posterior probabilities. Nodes with posterior probability < 0.5 are unresolved.

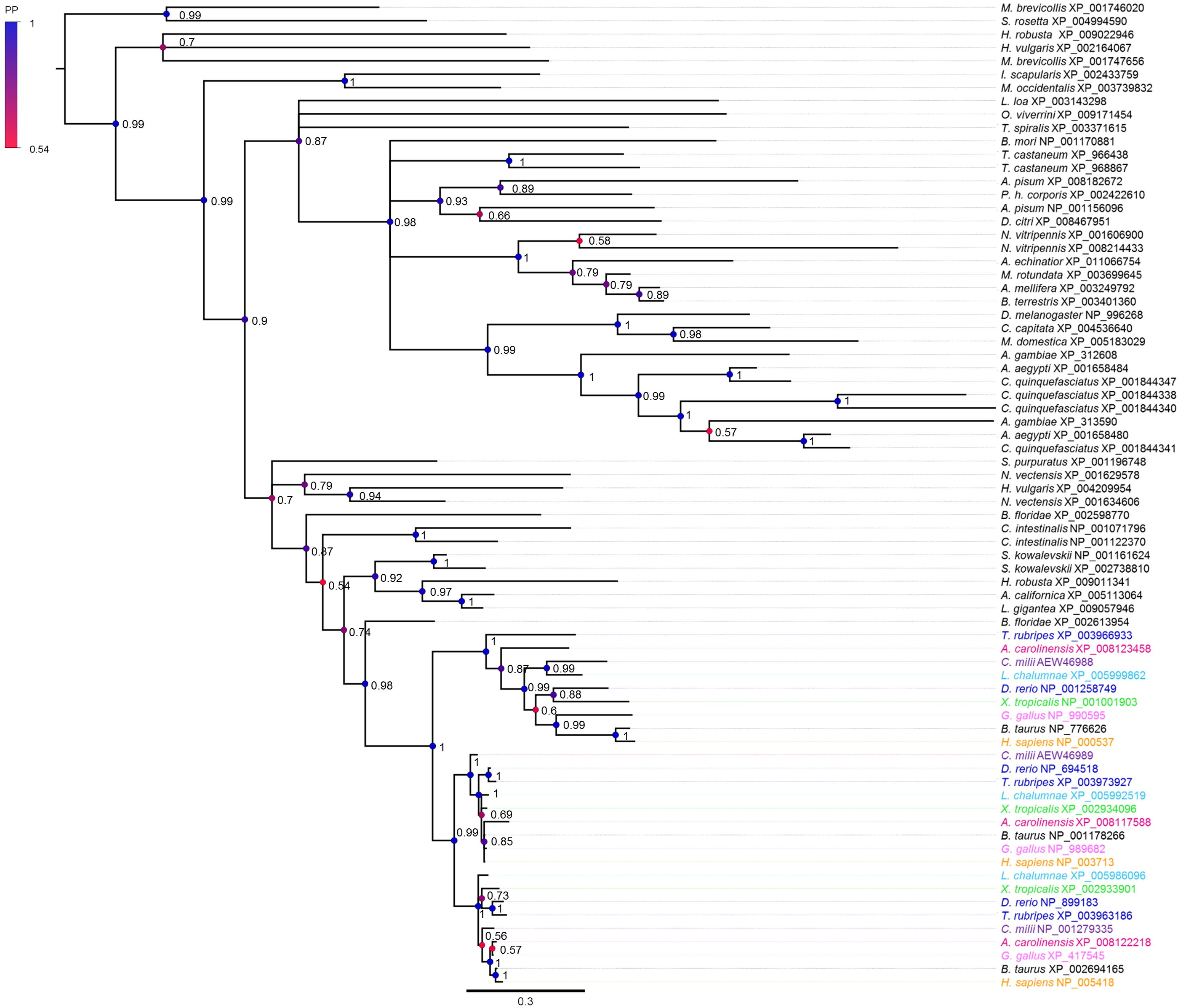
PP
1
0.54
M. brevicollis XP_001746020
S. rosetta XP_004994590
H. robusta XP_009022946
H. vulgaris XP_002164067
M. brevicollis XP_001747656
I. scapularis XP_002433759
M. occidentalis XP_003739832
L. loa XP_003143298
O. viverrini XP_009171454
T. spiralis XP_003371615
B. mori NP_001170881
T. castaneum XP_966438
T. castaneum XP_968867
A. pisum XP_008182672
P. h. corporis XP_002422610
A. pisum NP_001156096
D. citri XP_008467951
N. vitripennis XP_001606900
N. vitripennis XP_008214433
A. echinatior XP_011066754
M. rotundata XP_003699645
A. mellifera XP_003249792
B. terrestris XP_003401360
D. melanogaster NP_996268
C. capitata XP_004536640
M. domestica XP_005183029
A. gambiae XP_312608
A. aegypti XP_001658484
C. quinquefasciatus XP_001844347
C. quinquefasciatus XP_001844338
C. quinquefasciatus XP_001844340
A. gambiae XP_313590
A. aegypti XP_001658480
C. quinquefasciatus XP_001844341
S. purpuratus XP_001196748
N. vectensis XP_001629578
H. vulgaris XP_004209954
N. vectensis XP_001634606
B. floridae XP_002598770
C. intestinalis NP_001071796
C. intestinalis NP_001122370
S. kowalevskii NP_001161624
S. kowalevskii XP_002738810
H. robusta XP_009011341
A. californica XP_005113064
L. gigantea XP_009057946
B. floridae XP_002613954
T. rubripes XP_003966933
A. carolinensis XP_008123458
C. milii AEW46988
L. chalumnae XP_005999862
D. rerio NP_001258749
X. tropicalis NP_001001903
G. gallus NP_990595
B. taurus NP_776626
H. sapiens NP_000537
C. milii AEW46989
D. rerio NP_694518
T. rubripes XP_003973927
L. chalumnae XP_005992519
X. tropicalis XP_002934096
A. carolinensis XP_008117588
B. taurus NP_001178266
G. gallus NP_989682
H. sapiens NP_003713
L. chalumnae XP_005986096
X. tropicalis XP_002933901
D. rerio NP_899183
T. rubripes XP_003963186
C. milii NP_001279335
A. carolinensis XP_008122218
G. gallus XP_417545
B. taurus XP_002694165
H. sapiens NP_005418
0.3
