# Supplementary material for: Functional Diversification after Gene Duplication: Paralog Specific Regions of Structural Disorder and Phosphorylation in p53, p63, and p73
Source: PLoS One. 2016 Mar 22;11(3):e0151961. doi: 10.1371/journal.pone.0151961 (PMC4803236; doi:10.1371/journal.pone.0151961)
Supplement: S2 Fig — (A) Circular representations of p53 DNA-based phylogeny and (B) its corresponding full-protein-based phylogeny. These consensus trees were obtained with MrBayes 3.2.2 after sampling trees for 15 million generations with the default burn-in phase (discarding the first 25% of trees) and using the 50% majority rule. Node circles show posterior probabilities ranging from 0.5 in red to 1 in white. Here proteins were colored by clade (p53 in grey, p63 in blue and p73 in green) with tip labels following the color guide from Fig 2. Figure generated with FigTree (http://tree.bio.ed.ac.uk/software/figtree/). (PDF) [file pone.0151961.s002.pdf]

Supplementary material

S2 Fig. p53 Phylogenies for 301 Vertebrates Proteins. (A) Circular representations of p53 DNA-based phylogeny and (B) its corresponding full-protein-based phylogeny. These consensus trees were obtained with MrBayes 3.2.2 after sampling trees for 15 million generations with the default burn-in phase (discarding the first 25% of trees) and using the 50% majority rule. Node circles show posterior probabilities ranging from 0.5 in red to 1 in white. Here proteins were colored by clade (p53 in grey, p63 in blue and p73 in green) with tip labels following the color guide from Fig 2. Figure generated with FigTree (http://tree.bio.ed.ac.uk/software/figtree/).

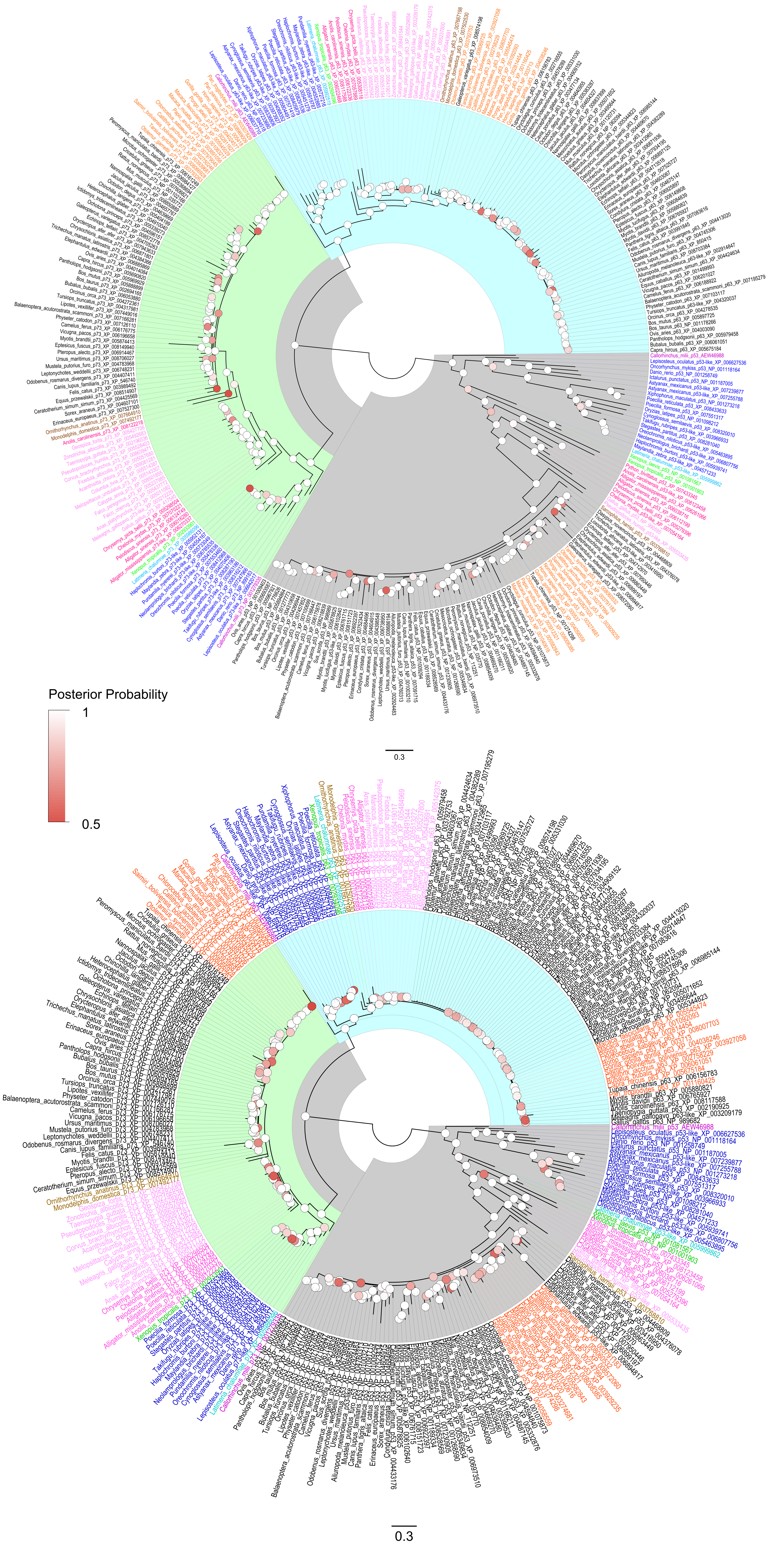
Posterior Probability
1
0.5
0.3
0.3
