# Supplementary material for: Functional Diversification after Gene Duplication: Paralog Specific Regions of Structural Disorder and Phosphorylation in p53, p63, and p73
Source: PLoS One. 2016 Mar 22;11(3):e0151961. doi: 10.1371/journal.pone.0151961 (PMC4803236; doi:10.1371/journal.pone.0151961)
Supplement: S3 Fig — (A) Heat map showing Pfam domain predictions per protein into their corresponding multiple sequence alignment sites (rows show protein hits; columns show alignment positions; sites that belong to Pfam_A domains are colored, green; linkers between domains, white; gaps in the alignment, grey), all in the context of the p53 DNA-based phylogeny with tip labels colored according to the color guide. (B) In addition, individual domain architectures (labeled and colored as shown in Pfam domains box) were also included to highlight their actual lengths enforcing missing or broken domains. Figure generated with iTOL [63]. (PDF) [file pone.0151961.s003.pdf]

Supplementary material

S3 Fig. Domain Composition in Vertebrate Proteins. (A) Heat map showing Pfam domain predictions per protein into their corresponding multiple sequence alignment sites (rows show protein hits; columns show alignment positions; sites that belong to Pfam_A domains are colored, green; linkers between domains, white; gaps in the alignment, grey), all in the context of the p53 DNA-based phylogeny with tip labels colored according to the color guide. (B) In addition, individual domain architectures (labeled and colored as shown in Pfam domains box) were also included to highlight their actual lengths enforcing missing or broken domains. Figure generated with iTOL (Letunic I, Bork P (2011) Interactive Tree Of Life v2: online annotation and display of phylogenetic trees made easy. Nucleic Acids Res 39: W475–W478).

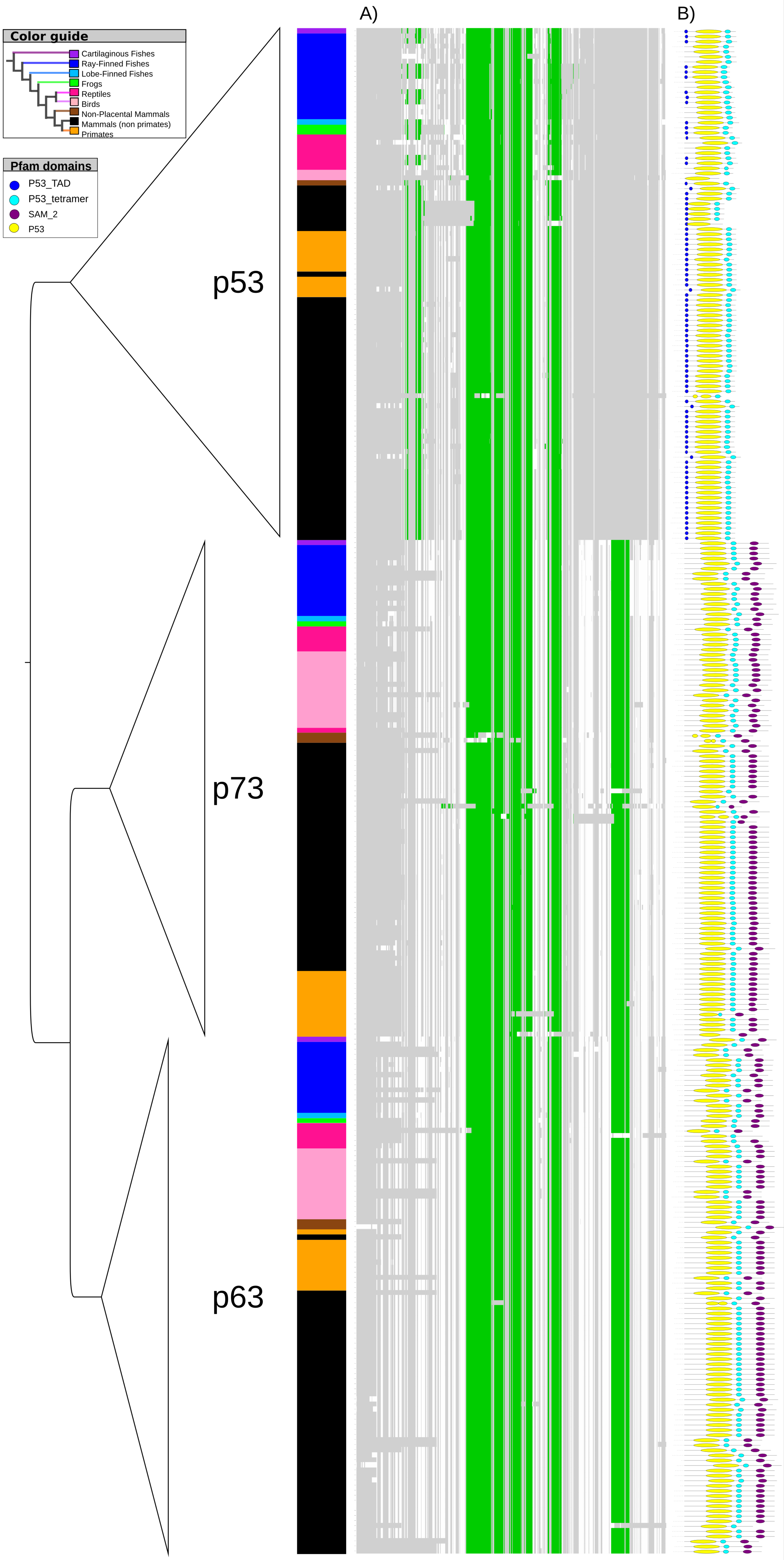

Color guide
Cartilaginous Fishes
Ray-Finned Fishes
Lobe-Finned Fishes
Frogs
Reptiles
Birds
Non-Placental Mammals
Mammals (non primates)
Primates
Pfam domains
P53_TAD
P53_tetramer
SAM_2
P53
A)
B)
p53
p73
p63
