# Supplementary material for: Functional Diversification after Gene Duplication: Paralog Specific Regions of Structural Disorder and Phosphorylation in p53, p63, and p73
Source: PLoS One. 2016 Mar 22;11(3):e0151961. doi: 10.1371/journal.pone.0151961 (PMC4803236; doi:10.1371/journal.pone.0151961)
Supplement: S4 Fig — Distribution of structural disorder (grey) and order (blue) in full-length proteins and in p53 DBD domains sorted by p53 DNA-based phylogenetic tree with tip labels following the color guide. Furthermore, individual domain architectures (labeled and colored as shown in Pfam domains box) were also included. Figure generated with iTOL [63]. (PDF) [file pone.0151961.s004.pdf]

Supplementary material

S4 Fig. Structural Disorder Fractions in Vertebrate Proteins. Distribution of structural disorder (grey) and order (blue) in full-length proteins and in p53 DBD domains sorted by p53 DNA-based phylogenetic tree with tip labels following the color guide. Furthermore, individual domain architectures (labeled and colored as shown in Pfam domains box) were also included. Figure generated with iTOL (Letunic I, Bork P (2011) Interactive Tree Of Life v2: online annotation and display of phylogenetic trees made easy. Nucleic Acids Res 39: W475–W478).

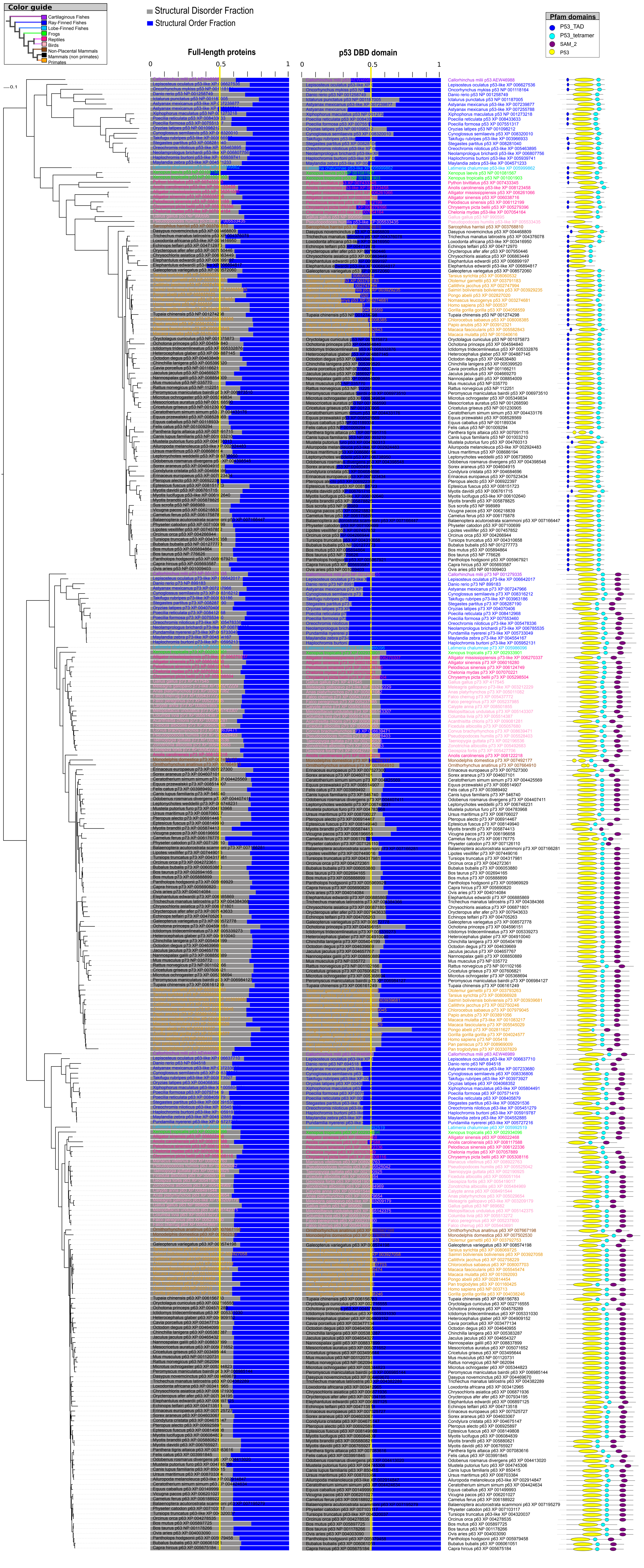

Color guide
Cartilaginous Fishes
Ray-Finned Fishes
Lobe-Finned Fishes
Frogs
Reptiles
Birds
Non-Placental Mammals
Mammals (non primates)
Primates
Structural Disorder Fraction
Structural Order Fraction
Full-length proteins
p53 DBD domain
Pfam domains
P53_TAD
P53_tetramer
SAM_2
P53
