# Supplementary material for: Functional Diversification after Gene Duplication: Paralog Specific Regions of Structural Disorder and Phosphorylation in p53, p63, and p73
Source: PLoS One. 2016 Mar 22;11(3):e0151961. doi: 10.1371/journal.pone.0151961 (PMC4803236; doi:10.1371/journal.pone.0151961)
Supplement: S5 Fig — Scatter plot of the p53 DBD structural disorder percentage vs. the number of Pfam domains per protein from 74 hits, including invertebrates and vertebrates proteins. There is a positive correlation between these two variables (Pearson correlation coefficient R = 0.64, R2 = 0.41, and p-value < 0.05, concluding that linear correlation different to 0 is statistically significant). (PDF) [file pone.0151961.s005.pdf]

Supplementary material

S5 Fig. p53 DBD Structural Disorder Content Increases with the Number of Domains. Scatter plot of the p53 DBD structural disorder percentage vs. the number of Pfam domains per protein from 74 hits, including invertebrates and vertebrates proteins. There is a positive correlation between these two variables (Pearson correlation coefficient R=0.64, R2=0.41, and p-value < 0.05, concluding that linear correlation different to 0 is statistically significant).

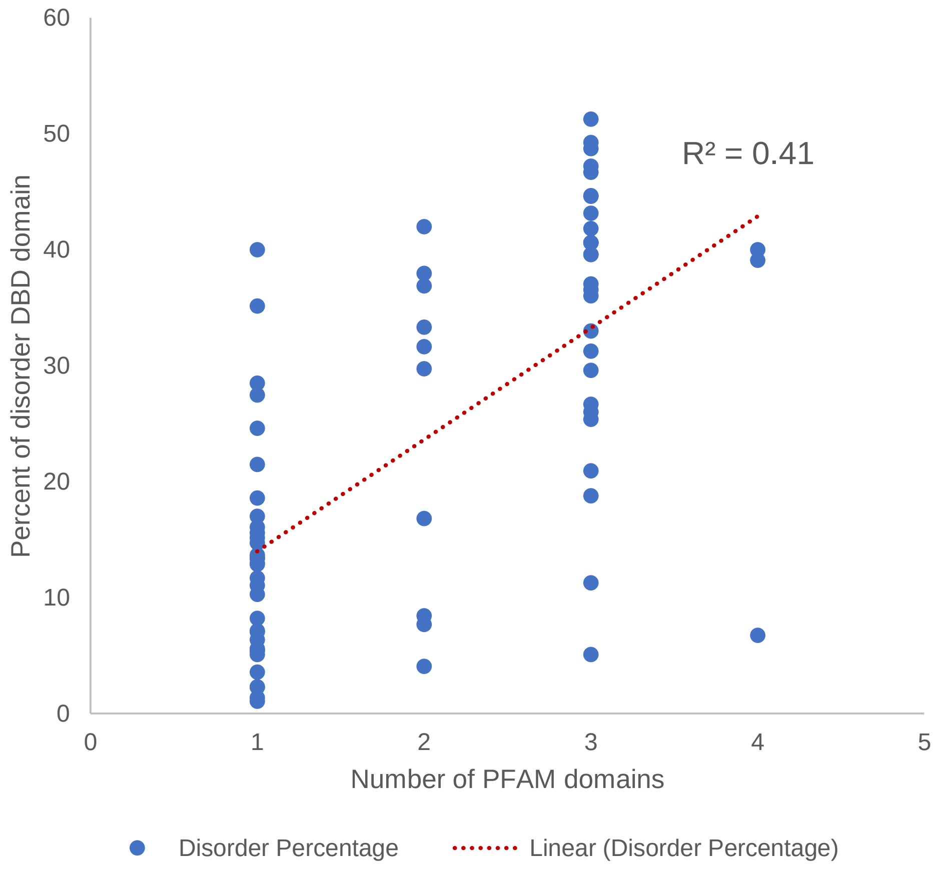

R² = 0.41
Percent of disorder DBD domain
Number of PFAM domains
0
10
20
30
40
50
60
0
1
2
3
4
5
Disorder Percentage
Linear (Disorder Percentage)
