# Supplementary material for: Functional Diversification after Gene Duplication: Paralog Specific Regions of Structural Disorder and Phosphorylation in p53, p63, and p73
Source: PLoS One. 2016 Mar 22;11(3):e0151961. doi: 10.1371/journal.pone.0151961 (PMC4803236; doi:10.1371/journal.pone.0151961)
Supplement: S6 Fig — Boxplots showing the fractions of serine, threonine and tyrosine residues per protein per clade compared to the fractions of sites predicted to be phosphorylated by NetPhos [25] using a 0.75 cut-off. Significance analysis was carried out using non-parametric tests (Kruskal Wallis test for the comparison of 3 or more samples and Mann-Whitney U test with Bonferroni correction for the pairwise analysis). Differences in means are statistically significant (p-values << 0.05), except for the p53-p63 comparison of predicted phosphorylation factions (p-value = 1). (PDF) [file pone.0151961.s006.pdf]

Supplementary material

S6 Fig. Differential Distribution of Protein Phosphorylations per Clade. Boxplots showing the fractions of serine, threonine and tyrosine residues per protein per clade compared to the fractions of sites predicted to be phosphorylated by NetPhos (Blom N, Gammeltoft S, Brunak S (1999) Sequence and structure-based prediction of eukaryotic protein phosphorylation sites. J Mol Biol 294: 1351–1362) using a 0.75 cut-off. Significance analysis was carried out using non-parametric tests (Kruskal Wallis test for the comparison of 3 or more samples and Mann-Whitney U test with Bonferroni correction for the pairwise analysis). Differences in means are statistically significant (p-values << 0.05), except for the p53-p63 comparison of predicted phosphorylation factions (p-value = 1).

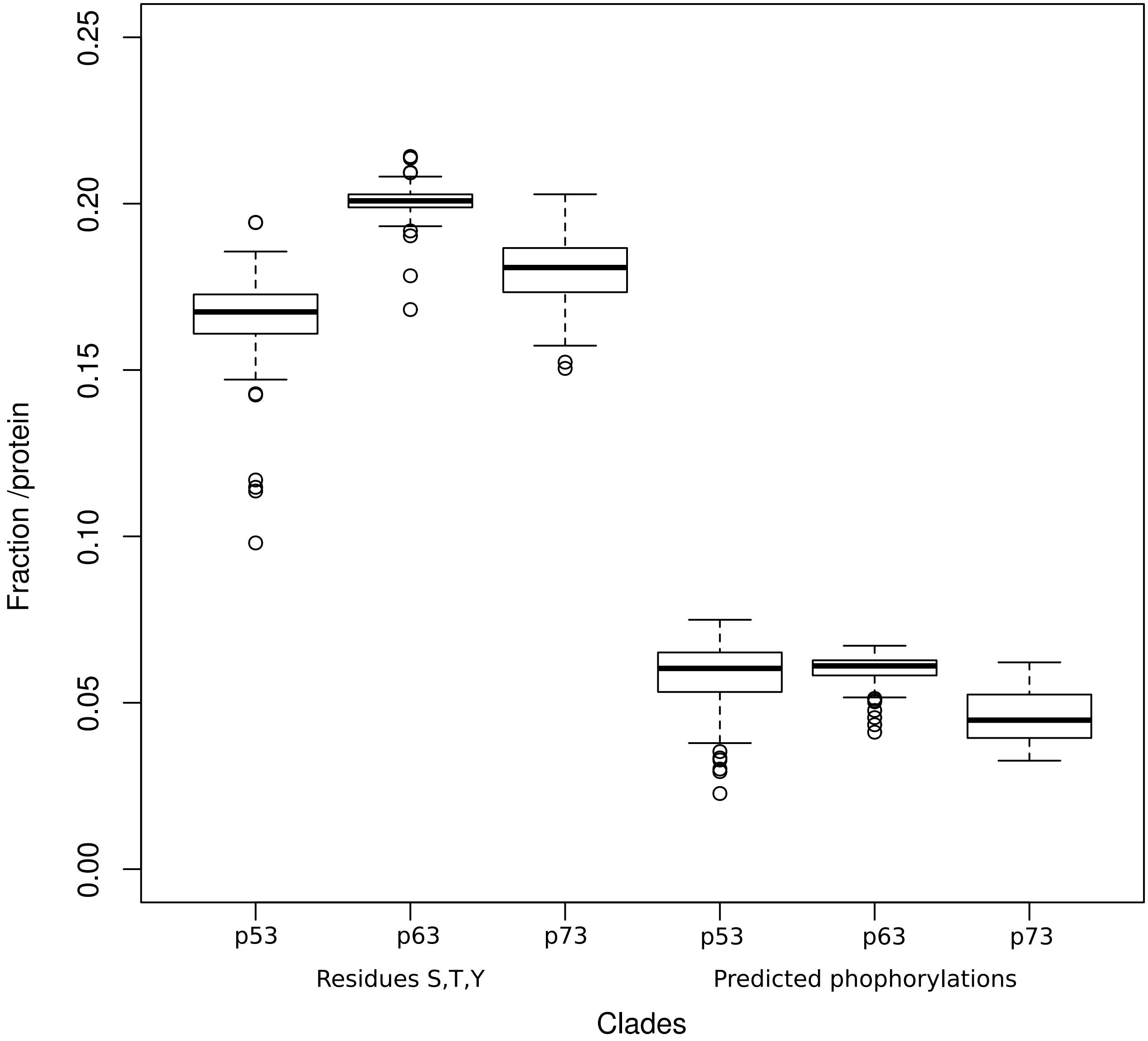

0.00
0.05
0.10
0.15
0.20
0.25
Fraction /protein
p53
p63
p73
p53
p63
p73
Residues S,T,Y
Predicted phophorylations
Clades
