# Supplementary material for: Functional Diversification after Gene Duplication: Paralog Specific Regions of Structural Disorder and Phosphorylation in p53, p63, and p73
Source: PLoS One. 2016 Mar 22;11(3):e0151961. doi: 10.1371/journal.pone.0151961 (PMC4803236; doi:10.1371/journal.pone.0151961)
Supplement: S8 Fig — Combined profiles of normalized evolutionary rates per aligned site for family and clades (vertebrates set) comparing amino acid substitutions (SEQ) with (A) secondary structure elements-loop transitions (SLT) and (B) phosphorylation transitions (PT). Grey shaded areas delimitate Pfam [66] domain regions. (PDF) [file pone.0151961.s008.pdf]

Supplementary material

S8 Fig. Comparison of SLT and PT with SEQ rates. Combined profiles of normalized evolutionary rates per aligned site for family and clades (vertebrates set) comparing amino acid substitutions (SEQ) with (A) secondary structure elements-loop transitions (SLT) and (B) phosphorylation transitions (PT). Grey shaded areas delimitate Pfam domain regions.

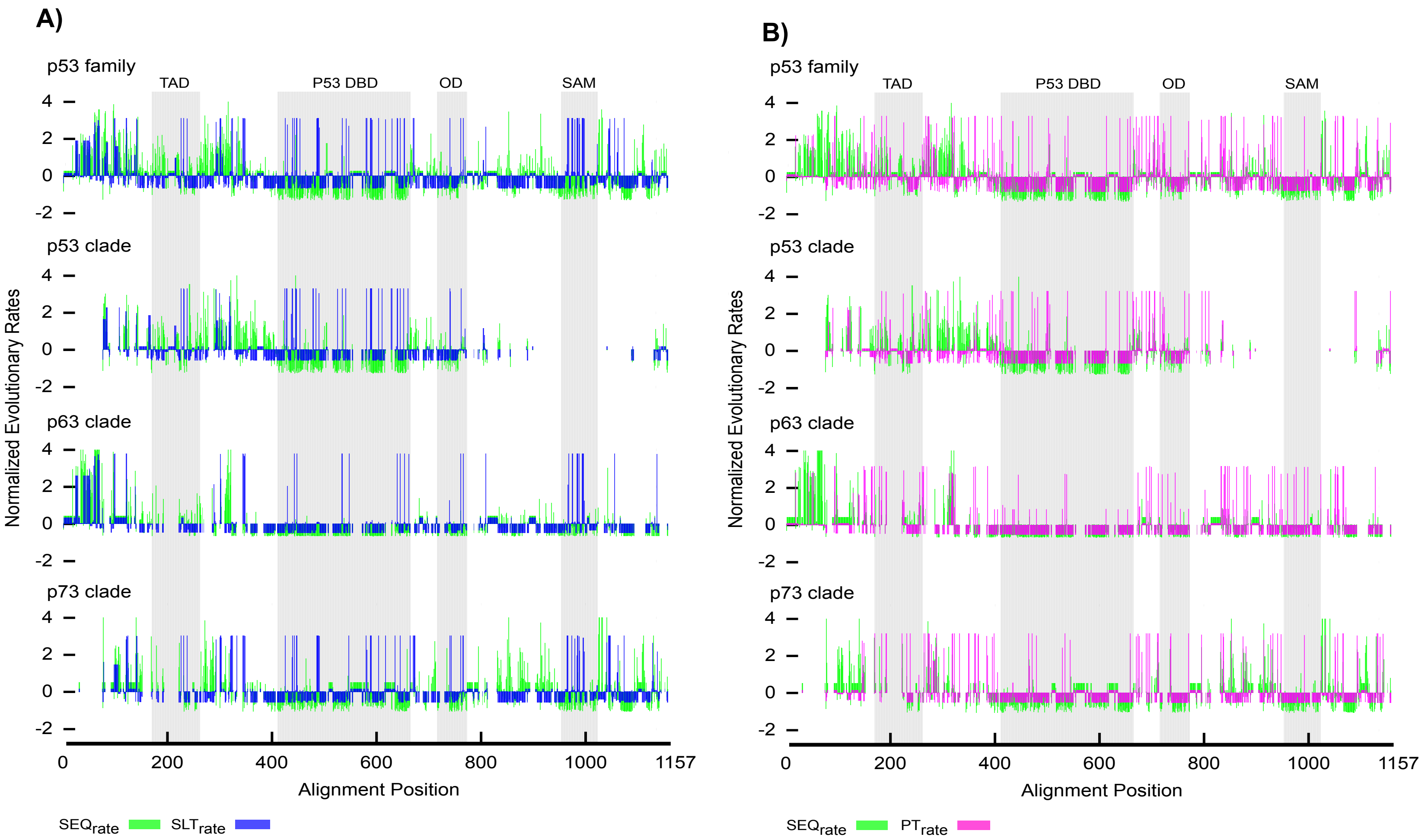

A)
B)
p53 family
p53 clade
p63 clade
p73 clade
TAD
P53 DBD
OD
SAM
Normalized Evolutionary Rates
Alignment Position
0
200
400
600
800
1000
1157
4
2
0
-2
SEQrate
SLTrate
PTrate
