# Supplementary material for: Functional Diversification after Gene Duplication: Paralog Specific Regions of Structural Disorder and Phosphorylation in p53, p63, and p73
Source: PLoS One. 2016 Mar 22;11(3):e0151961. doi: 10.1371/journal.pone.0151961 (PMC4803236; doi:10.1371/journal.pone.0151961)
Supplement: S9 Fig — Heat map for structural traits plotted in the order of the p53 DNA-based phylogenetic tree context, showing p53 protein names as boxes colored according to the color guide in Fig 2. These heat maps are showing sequence-based predictions mapped to their corresponding residue sites in the multiple sequence alignment, after removing empty columns (i.e. columns fully gapped in the p53 clade) for this subset: (A) continuous structural disorder propensities by IUPred [15,62] with a color gradient from blue to white to red mirroring the disorder propensity gradient from low (blue) to high (red), with white being the boundary between order and disorder (remaining alignment gaps are colored in grey). (B) secondary structure predictions by PSIPRED [24,64] displaying 3 states loop (white), alpha helix (purple) and beta strand (yellow), and C) sites predicted to be phosphorylated by NetPhos [25] using a 0.75 cut-off (red). On top of these heat maps, normalized evolutionary rates per site are shown for amino acid sequence (SEQ) in green [26] vs. binary traits [27] of disorder-order transitions (DOT) in orange (upper left), secondary structure elements—loop transitions (SLT) in blue (upper center), and phosphorylation transitions (PT) in pink (upper right). All evolutionary rates were normalized with a mean of zero and standard deviation of 1: negative rates for slow evolving sites and positive rates for fast evolving sites. Grey shaded areas delimitate Pfam domain regions. (PDF) [file pone.0151961.s009.pdf]

Supplementary material

S9 Fig. p53 clade in detail: graphical representation of sequence-based predictions. Heat map for structural traits plotted in the order of the p53 DNA-based phylogenetic tree context, showing p53 protein names as boxes colored according to the color guide in Fig 2. These heat maps are showing sequence-based predictions mapped to their corresponding residue sites in the multiple sequence alignment, after removing empty columns (i.e. columns fully gapped in the p53 clade) for this subset: (A) continuous structural disorder propensities by IUPred [1] with a color gradient from blue to white to red mirroring the disorder propensity gradient from low (blue) to high (red), with white being the boundary between order and disorder (remaining alignment gaps are colored in grey). (B) secondary structure predictions by PSIPRED [2] displaying 3 states loop (white), alpha helix (purple) and beta strand (yellow), and C) sites predicted to be phosphorylated by NetPhos [3] using a 0.75 cut-off (red). On top of these heat maps, normalized evolutionary rates per site are shown for amino acid sequence (SEQ) in green [4] vs. binary traits [5] of disorder-order transitions (DOT) in orange (upper left), secondary structure elements - loop transitions (SLT) in blue (upper center), and phosphorylation transitions (PT) in pink (upper right). All evolutionary rates were normalized with a mean of zero and standard deviation of 1: negative rates for slow evolving sites and positive rates for fast evolving sites. Grey shaded areas delimitate Pfam domain regions [6].

References

1. Dosztányi Z, Csizmok V, Tompa P, Simon I (2005) IUPred: web server for the prediction of intrinsically unstructured regions of proteins based on estimated energy content. Bioinformatics 21: 3433–3434.

2. McGuffin LJ, Bryson K, Jones DT (2000) The PSIPRED protein structure prediction server. Bioinformatics 16: 404–405.

3. Blom N, Gammeltoft S, Brunak S (1999) Sequence and structure-based prediction of eukaryotic protein phosphorylation sites. J Mol Biol 294: 1351–1362.

4. Pupko T, Bell RE, Mayrose I, Glaser F, Ben-Tal N (2002) Rate4Site: an algorithmic tool for the identification of functional regions in proteins by surface mapping of evolutionary determinants within their homologues. Bioinformatics 18: S71–S77.

5. Cohen O, Ashkenazy H, Belinky F, Huchon D, Pupko T (2010) GLOOME: gain loss mapping engine. Bioinformatics 26: 2914–2915.

6. Finn RD, Bateman A, Clements J, Coggill P, Eberhardt RY, et al. (2014) Pfam: the protein families database. Nucleic Acids Res 42: D222–D230. doi:10.1093/nar/gkt1223.

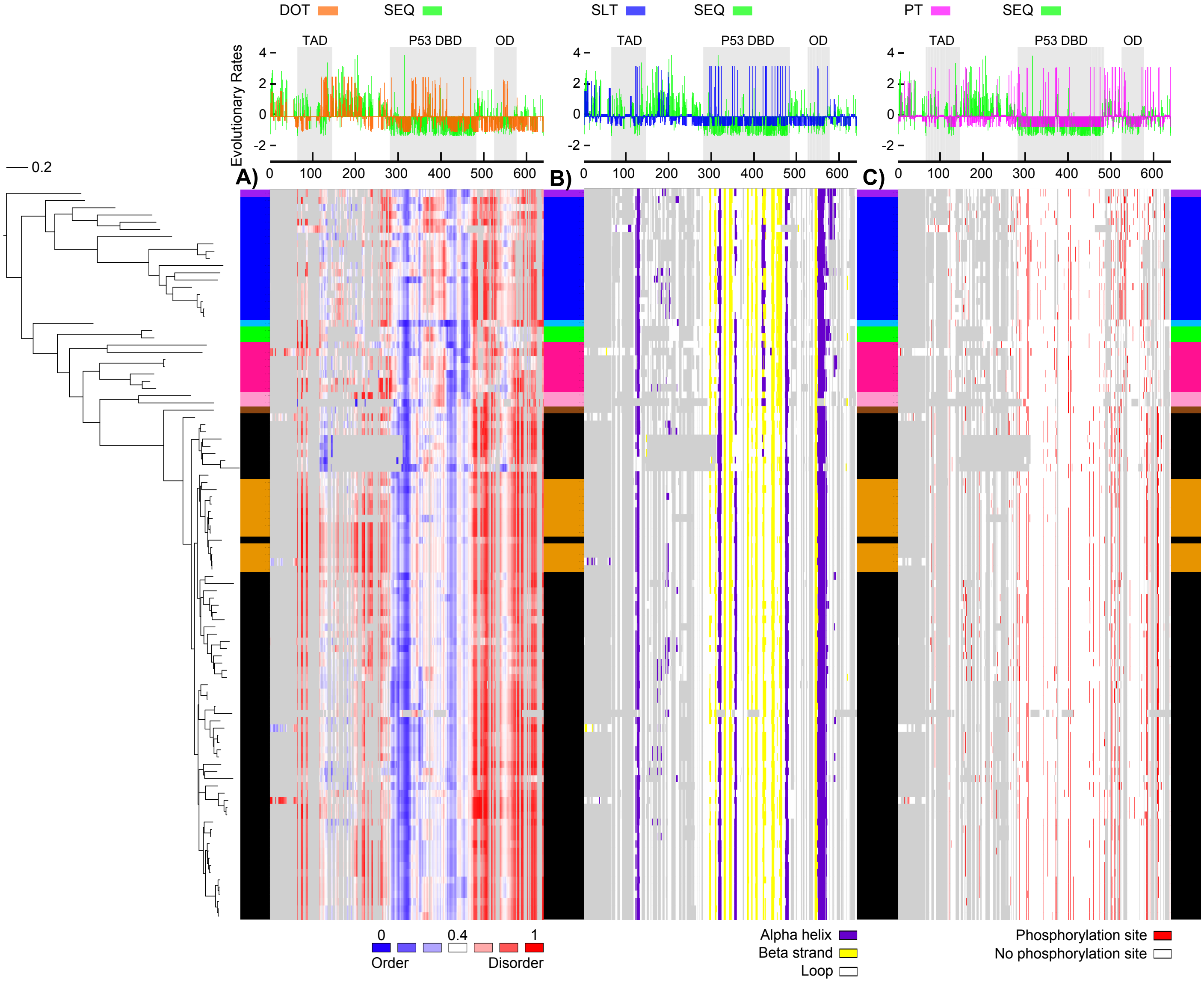
DOT
SEQ
SLT
SEQ
PT
SEQ
TAD
P53 DBD
OD
Evolutionary Rates
4
2
0
-2
0
100
200
300
400
500
600
0.2
A)
B)
C)
0
0.4
1
Order
Disorder
Alpha helix
Beta strand
Loop
Phosphorylation site
No phosphorylation site
