# Supplementary material for: Functional Diversification after Gene Duplication: Paralog Specific Regions of Structural Disorder and Phosphorylation in p53, p63, and p73
Source: PLoS One. 2016 Mar 22;11(3):e0151961. doi: 10.1371/journal.pone.0151961 (PMC4803236; doi:10.1371/journal.pone.0151961)
Supplement: S1 Table — Accession numbers for the vertebrate datasets (i) and (ii). (PDF) [file pone.0151961.s010.pdf]

Supplementary material

S1 Table Accession numbers for the vertebrate datasets (i) and (ii)

#Genbank (June 2015):

Organism	mRNAcodes	GeneIDs	ProteinCodes	Product
Xenopus_laevis	NM_001088098	397926	NP_001081567.1	cellular_tumor_antigen_p53
Loxodonta_africana	XM_003412917	100657932	XP_003412965.1	tumor_protein_63_isoform_X1
Loxodonta_africana	XM_003416902	100663725	XP_003416950.1	cellular_tumor_antigen_p53
Vicugna_pacos	XM_006196596	102538948	XP_006196658.1	tumor_protein_p73_isoform_X1
Vicugna_pacos	XM_006200965	102524910	XP_006201027.1	tumor_protein_63_isoform_X1
Vicugna_pacos	XM_006218777	102530959	XP_006218839.1	cellular_tumor_antigen_p53
Poecilia_formosa	XM_007551255	103137501	XP_007551317.1	cellular_tumor_antigen_p53
Poecilia_formosa	XM_007553398	103138976	XP_007553460.1	tumor_protein_p73_isoform_X2
Poecilia_formosa	XM_007571357	103151349	XP_007571419.1	tumor_protein_63_isoform_X1
Alligator_mississippiensis	XM_006261004	102570618	XP_006261066.1	cellular_tumor_antigen_p53_isoform_X2
Alligator_mississippiensis	XM_006270275	102575914	XP_006270337.1	tumor_protein_p73-like
Corvus_brachyrhynchos	XM_008641249	103622329	XP_008639471.1	LOW_QUALITY_PROTEIN:_tumor_protein_p73
Ochotona_princeps	XM_004578232	101534187	XP_004578289.1	tumor_protein_63
Ochotona_princeps	XM_004594783	101528346	XP_004594840.1	cellular_tumor_antigen_p53
Ochotona_princeps	XM_004596094	101519640	XP_004596151.1	tumor_protein_p73_isoform_X2
Panthera_tigris	XM_007083554	102950776	XP_007083616.1	tumor_protein_63_isoform_X2
Panthera_tigris	XM_007091653	102962571	XP_007091715.1	cellular_tumor_antigen_p53
Calypte_anna	XM_008493322	103528049	XP_008491544.1	tumor_protein_63_isoform_X1
Calypte_anna	XM_008503633	103537360	XP_008501855.1	tumor_protein_p73_isoform_X1
Balaenoptera_acutorostrata	XM_007166219	103002230	XP_007166281.1	tumor_protein_p73_isoform_X1
Balaenoptera_acutorostrata	XM_007166385	103003426	XP_007166447.1	cellular_tumor_antigen_p53_isoform_X1
Balaenoptera_acutorostrata	XM_007195217	103005802	XP_007195279.1	tumor_protein_63_isoform_X1
Stegastes_partitus	XM_008282818	103358022	XP_008281040.1	cellular_tumor_antigen_p53
Stegastes_partitus	XM_008288968	103362580	XP_008287190.1	tumor_protein_p73_isoform_X2
Stegastes_partitus	XM_008293314	103365775	XP_008291536.1	tumor_protein_63-like_isoform_X1
Eptesicus_fuscus	XM_008151586	103295159	XP_008149808.1	tumor_protein_63_isoform_X1
Eptesicus_fuscus	XM_008151718	103295286	XP_008149940.1	tumor_protein_p73_isoform_X1
Eptesicus_fuscus	XM_008153501	103296897	XP_008151723.1	cellular_tumor_antigen_p53
Pteropus_alecto	XM_006922335	102889604	XP_006922397.1	cellular_tumor_antigen_p53
Pteropus_alecto	XM_006925835	102889462	XP_006925897.1	tumor_protein_63_isoform_X1
Pteropus_alecto	XM_006914405	102895499	XP_006914467.1	tumor_protein_p73_isoform_X1
Saimiri_boliviensis	XM_003929186	101043647	XP_003929235.1	cellular_tumor_antigen_p53
Bos_mutus	XM_005888837	102267267	XP_005888899.1	tumor_protein_p73_isoform_X1
Bos_mutus	XM_005894802	102272370	XP_005894864.1	cellular_tumor_antigen_p53
Bos_mutus	XM_005897663	102283023	XP_005897725.1	tumor_protein_63_isoform_X3
Tursiops_truncatus	XM_004310610	101319146	XP_004310658.1	cellular_tumor_antigen_p53_isoform_1
Tursiops_truncatus	XM_004317933	101335289	XP_004317981.1	tumor_protein_p73_isoform_1
Tursiops_truncatus	XM_004319989	101319969	XP_004320037.1	tumor_protein_63-like_isoform_1
Myotis_brandtii	XM_005874351	102240853	XP_005874413.1	tumor_protein_p73
Myotis_brandtii	XM_005878763	102244768	XP_005878825.1	cellular_tumor_antigen_p53
Myotis_brandtii	XM_005880759	102244990	XP_005880821.1	tumor_protein_63_isoform_X1
Melopsittacus_undulatus	XM_005142318	101873384	XP_005142375.1	tumor_protein_63_isoform_X1
Melopsittacus_undulatus	XM_005143250	101873559	XP_005143307.1	tumor_protein_p73_isoform_X2
Python_bivittatus	XM_007433283	103061365	XP_007433345.1	cellular_tumor_antigen_p53
Haplochromis_burtoni	XM_005919725	102293825	XP_005919787.1	tumor_protein_63-like_isoform_X5
Haplochromis_burtoni	XM_005939679	102296334	XP_005939741.1	cellular_tumor_antigen_p53-like_isoform_X3
Haplochromis_burtoni	XM_005952069	102297399	XP_005952131.1	tumor_protein_p73-like_isoform_X3
Elephantulus_edwardii	XM_006885807	102848919	XP_006885869.1	tumor_protein_p73_isoform_X1
Elephantulus_edwardii	XM_006894755	102868163	XP_006894817.1	cellular_tumor_antigen_p53-like
Elephantulus_edwardii	XM_006897063	102870614	XP_006897125.1	tumor_protein_63_isoform_X1
Elephantulus_edwardii	XM_006899135	102863330	XP_006899197.1	cellular_tumor_antigen_p53
Chrysochloris_asiatica	XM_006863387	102835323	XP_006863449.1	cellular_tumor_antigen_p53
Chrysochloris_asiatica	XM_006871739	102842286	XP_006871801.1	tumor_protein_p73_isoform_X1
Chrysochloris_asiatica	XM_006871874	102824152	XP_006871936.1	tumor_protein_63_isoform_X1
Bos_taurus	NM_174201	281542	NP_776626.1	cellular_tumor_antigen_p53
Bos_taurus	NM_001191337	615335	NP_001178266.1	tumor_protein_63
Bos_taurus	XM_002694119	515105	XP_002694165.1	tumor_protein_p73_isoform_X4
Ictalurus_punctatus	NM_001200076	100304476	NP_001187005.1	cellular_tumor_antigen_p53
Gallus_gallus	NM_204351	374269	NP_989682.1	tumor_protein_63
Gallus_gallus	NM_205264	396200	NP_990595.1	cellular_tumor_antigen_p53
Gallus_gallus	XM_417545	419382	XP_417545.3	tumor_protein_p73_isoform_X5
Pan_troglodytes	XM_001160425	460930	XP_001160425.1	tumor_protein_63_isoform_X4

Alligator_sinensis	XM_006016218	102384735	XP_006016280.1	tumor_protein_p73
Alligator_sinensis	XM_006022406	102376691	XP_006022468.1	tumor_protein_63_isoform_X1
Alligator_sinensis	XM_006038654	102376594	XP_006038716.1	cellular_tumor_antigen_p53
Cricetulus_griseus	NM_001243976	100682525	NP_001230905.1	cellular_tumor_antigen_p53
Cricetulus_griseus	XM_003495596	100772309	XP_003495644.1	tumor_protein_63_isoform_X1
Cricetulus_griseus	XM_007608631	100774060	XP_007606821.1	tumor_protein_p73_isoform_X2
Pelodiscus_sinensis	XM_006112137	102448135	XP_006112199.1	cellular_tumor_antigen_p53_isoform_X2
Pelodiscus_sinensis	XM_006122274	102445069	XP_006122336.1	tumor_protein_63
Pelodiscus_sinensis	XM_006124687	102457368	XP_006124749.1	tumor_protein_p73
Tupaia_chinensis	XM_006156721	102489678	XP_006156783.1	tumor_protein_63_isoform_X1
Tupaia_chinensis	XM_006161187	102492504	XP_006161249.1	tumor_protein_p73
Tupaia_chinensis	NM_001287369	102503238	NP_001274298.1	tumor_protein_p53
Pantholops_hodgsonii	XM_005967859	102330970	XP_005967921.1	cellular_tumor_antigen_p53_isoform_X1
Pantholops_hodgsonii	XM_005969867	102327041	XP_005969929.1	tumor_protein_p73_isoform_X1
Pantholops_hodgsonii	XM_005979396	102341828	XP_005979458.1	tumor_protein_63_isoform_X5
Latimeria_chalumnae	XM_005986034	102364155	XP_005986096.1	tumor_protein_p73
Latimeria_chalumnae	XM_005992457	102352568	XP_005992519.1	tumor_protein_63_isoform_X1
Latimeria_chalumnae	XM_005999800	102366539	XP_005999862.1	cellular_tumor_antigen_p53-like_isoform_X3
Ficedula_albicollis	XM_005051107	101821339	XP_005051164.1	tumor_protein_63_isoform_X1
Ficedula_albicollis	XM_005057623	101820371	XP_005057680.1	tumor_protein_p73_isoform_X2
Macaca_fascicularis	XM_005544972	102124774	XP_005545029.1	tumor_protein_p73_isoform_X2
Macaca_fascicularis	XM_005545417	102137688	XP_005545474.1	tumor_protein_63_isoform_X2
Macaca_fascicularis	XM_005582786	102135998	XP_005582843.1	cellular_tumor_antigen_p53_isoform_X1
Octodon_degus	XM_004638423	101589830	XP_004638480.1	cellular_tumor_antigen_p53_isoform_X1
Octodon_degus	XM_004639612	101591427	XP_004639669.1	tumor_protein_p73_isoform_X4
Octodon_degus	XM_004640898	101575887	XP_004640955.1	tumor_protein_63_isoform_X1
Canis_lupus	NM_001003210	403869	NP_001003210.1	cellular_tumor_antigen_p53
Canis_lupus	XM_546740	489620	XP_546740.3	tumor_protein_p73_isoformX3
Canis_lupus	XM_845322	488125	XP_850415.3	tumor_protein_63_isoformX2
Felis_catus	NM_001009294	493847	NP_001009294.1	cellular_tumor_antigen_p53
Felis_catus	XM_003989443	101088009	XP_003989492.1	tumor_protein_p73
Felis_catus	XM_003991796	101095124	XP_003991845.2	tumor_protein_63_isoform_X1
Mustela_putorius	XM_004745249	101680431	XP_004745306.1	tumor_protein_63_isoform_X1
Mustela_putorius	XM_004760256	101677794	XP_004760313.1	cellular_tumor_antigen_p53_isoform_X2
Mustela_putorius	XM_004783911	101679933	XP_004783968.1	tumor_protein_p73
Cavia_porcellus	NM_001172740	100379269	NP_001166211.1	cellular_tumor_antigen_p53
Cavia_porcellus	XM_003477086	100732143	XP_003477134.1	tumor_protein_63_isoformX1
Callorhinchus_milii	NM_001292406	103184209	NP_001279335.1	tumor_protein_p73
Sorex_araneus	XM_004603010	101537697	XP_004603067.1	tumor_protein_63_isoform_X1
Sorex_araneus	XM_004604858	101558045	XP_004604915.1	cellular_tumor_antigen_p53_isoform_X1
Sorex_araneus	XM_004607044	101543742	XP_004607101.1	tumor_protein_p73_isoform_X2
Ailuropoda_melanoleuca	XM_002914801	100479308	XP_002914847.2	tumor_protein_63_isoform_X1
Ailuropoda_melanoleuca	XM_002924437	100468516	XP_002924483.1	cellular_tumor_antigen_p53
Capra_hircus	XM_005675127	102190550	XP_005675184.1	tumor_protein_63_isoform_X3
Capra_hircus	XM_005690763	102184175	XP_005690820.1	LOW_QUALITY_PROTEIN:_tumor_protein_p73
Capra_hircus	XM_005693530	102169621	XP_005693587.1	cellular_tumor_antigen_p53
Mesocricetus_auratus	XM_005071595	101843398	XP_005071652.1	tumor_protein_63_isoform_X1
Mesocricetus_auratus	NM_001281661	101833915	NP_001268590.1	cellular_tumor_antigen_p53
Manacus_vitellinus	XM_008924515	103758266	XP_008922763.1	tumor_protein_63_isoform_X1
Monodelphis_domestica	XM_007492115	103106761	XP_007492177.1	tumor_protein_p73
Monodelphis_domestica	XM_007502468	100017464	XP_007502530.1	tumor_protein_63_isoform_X1
Anolis_carolinensis	XM_008119381	100562575	XP_008117588.1	tumor_protein_63
Anolis_carolinensis	XM_008124011	100554137	XP_008122218.1	tumor_protein_p73
Anolis_carolinensis	XM_008125251	103282543	XP_008123458.1	cellular_tumor_antigen_p53-like
Chlorocebus_sabaeus	XM_008009512	103241894	XP_008007703.1	LOW_QUALITY_PROTEIN:_tumor_protein_63
Chlorocebus_sabaeus	XM_008010194	103242323	XP_008008385.1	cellular_tumor_antigen_p53_isoform_X2
Chlorocebus_sabaeus	XM_007980854	103225731	XP_007979045.1	tumor_protein_p73_isoform_X1
Chelonia_mydas	XM_007070159	102933223	XP_007070221.1	tumor_protein_p73_isoform_X1
Chelonia_mydas	XM_007054102	102934531	XP_007054164.1	cellular_tumor_antigen_p53-like
Chelonia_mydas	XM_007057827	102935227	XP_007057889.1	tumor_protein_63_isoform_X2
Poecilia_reticulata	XM_008407657	103463932	XP_008405879.1	tumor_protein_63_isoform_X1
Poecilia_reticulata	XM_008414746	103467981	XP_008412968.1	tumor_protein_p73_isoform_X2
Poecilia_reticulata	XM_008435411	103480460	XP_008433633.1	cellular_tumor_antigen_p53
Equus_caballus	NM_001202405	100062044	NP_001189334.1	cellular_tumor_antigen_p53

Equus_caballus	XM_001499943	100059752	XP_001499993.3	tumor_protein_63_isoformX1
Mus_musculus	NM_011642	22062	NP_035772.2	tumor_protein_p73_isoform_a
Mus_musculus	NM_011640	22059	NP_035770.2	cellular_tumor_antigen_p53_isoform_a
Mus_musculus	NM_001127259	22061	NP_001120731.1	tumor_protein_63_isoform_a
Homo_sapiens	NM_003722	8626	NP_003713.3	tumor_protein_63_isoform_1
Homo_sapiens	NM_005427	7161	NP_005418.1	tumor_protein_p73_isoform_a
Homo_sapiens	NM_000546	7157	NP_000537.3	cellular_tumor_antigen_p53_isoform_a
Oryzias_latipes	NM_001104742	100049321	NP_001098212.1	cellular_tumor_antigen_p53
Oryzias_latipes	XM_004070358	100170633	XP_004070406.1	tumor_protein_p73_isoform_X1
Orcinus_orca	XM_004266896	101285670	XP_004266944.1	cellular_tumor_antigen_p53
Orcinus_orca	XM_004272313	101287226	XP_004272361.1	tumor_protein_p73
Orcinus_orca	XM_004278487	101287998	XP_004278535.1	tumor_protein_63_isoform_X1
Jaculus_jaculus	XM_004654270	101604054	XP_004654327.1	tumor_protein_63_isoform_X1
Jaculus_jaculus	XM_004657710	101595047	XP_004657767.1	tumor_protein_p73_isoform_X5
Jaculus_jaculus	XM_004669213	101609339	XP_004669270.1	cellular_tumor_antigen_p53_isoform_X1
Myotis_lucifugus	XM_006084777	102423348	XP_006084839.1	tumor_protein_63_isoform_X1
Myotis_lucifugus	XM_006102578	102431418	XP_006102640.1	cellular_tumor_antigen_p53-like
Chinchilla_lanigera	XM_005383230	102017241	XP_005383287.1	tumor_protein_63_isoform_X1
Chinchilla_lanigera	XM_005399463	102005455	XP_005399520.1	cellular_tumor_antigen_p53_isoform_X1
Chinchilla_lanigera	XM_005404142	102025470	XP_005404199.1	tumor_protein_p73_isoform_X1
Anas_platyrhynchos	XM_005011025	101797101	XP_005011082.1	tumor_protein_p73_isoform_X1
Anas_platyrhynchos	XM_005029597	101794454	XP_005029654.1	tumor_protein_63_isoform_X1
Geospiza_fortis	XM_005418960	102040828	XP_005419017.1	tumor_protein_63_isoform_X1
Geospiza_fortis	XM_005427649	102034695	XP_005427706.1	tumor_protein_p73_isoform_X1
Astyanax_mexicanus	XM_007233618	103030173	XP_007233680.1	tumor_protein_63-like_isoform_X1
Astyanax_mexicanus	XM_007239815	103046742	XP_007239877.1	cellular_tumor_antigen_p53-like
Astyanax_mexicanus	XM_007247904	103027594	XP_007247966.1	tumor_protein_p73_isoform_X2
Astyanax_mexicanus	XM_007255726	103037427	XP_007255788.1	cellular_tumor_antigen_p53-like
Myotis_davidii	XM_006765864	102758559	XP_006765927.1	tumor_protein_63_isoform_X1
Myotis_davidii	XM_006761652	102759012	XP_006761715.1	cellular_tumor_antigen_p53_isoform_X1
Heterocephalus_glaber	XM_004887088	101701769	XP_004887145.1	cellular_tumor_antigen_p53_isoform_X3
Heterocephalus_glaber	XM_004909095	101710600	XP_004909152.1	tumor_protein_63_isoform_X1
Heterocephalus_glaber	XM_004909983	101716466	XP_004910040.1	tumor_protein_p73_isoform_X3
Neolamprologus_brichardi	XM_006785472	102784606	XP_006785535.1	tumor_protein_p73-like
Neolamprologus_brichardi	XM_006807693	102776078	XP_006807756.1	cellular_tumor_antigen_p53-like_isoform_X1
Oreochromis_niloticus	XM_005478279	100694943	XP_005478336.1	tumor_protein_p73-like_isoform_X3
Oreochromis_niloticus	XM_005451222	100698132	XP_005451279.1	tumor_protein_63-like_isoform_X2
Oreochromis_niloticus	XM_005463838	100702587	XP_005463895.1	cellular_tumor_antigen_p53-like
Rattus_norvegicus	NM_001108696	362675	NP_001102166.1	tumor_protein_p73
Rattus_norvegicus	NM_030989	24842	NP_112251.2	cellular_tumor_antigen_p53
Rattus_norvegicus	NM_019221	246334	NP_062094.1	tumor_protein_63_isoform_a
Papio_anubis	XM_003891007	100998775	XP_003891056.1	tumor_protein_p73
Papio_anubis	XM_003912272	101006608	XP_003912321.1	cellular_tumor_antigen_p53
Orycteropus_afer	XM_007936004	103192917	XP_007934195.1	tumor_protein_63_isoform_X1
Orycteropus_afer	XM_007945442	103200856	XP_007943633.1	tumor_protein_p73_isoform_X1
Orycteropus_afer	XM_007952255	103206754	XP_007950446.1	cellular_tumor_antigen_p53
Odobenus_rosmarus	XM_004398491	101362427	XP_004398548.1	cellular_tumor_antigen_p53
Odobenus_rosmarus	XM_004407354	101377328	XP_004407411.1	tumor_protein_p73_isoform_X2
Odobenus_rosmarus	XM_004412963	101373970	XP_004413020.2	tumor_protein_63_isoform_X1
Falco_peregrinus	XM_005237743	101917750	XP_005237800.1	tumor_protein_63_isoform_X1
Falco_peregrinus	XM_005237928	101921570	XP_005237985.1	tumor_protein_p73_isoform_X1
Tarsius_syrichta	XM_008062341	103264637	XP_008060532.1	cellular_tumor_antigen_p53
Tarsius_syrichta	XM_008068737	103271276	XP_008066928.1	tumor_protein_p73_isoform_X4
Tarsius_syrichta	XM_008071534	103274066	XP_008069725.1	tumor_protein_63_isoform_X1
Sus_scrofa	NM_213824	397276	NP_998989.3	cellular_tumor_antigen_p53
Ornithorhynchus_anatinus	XM_007669008	100081838	XP_007667198.1	tumor_protein_63_isoform_X2
Ornithorhynchus_anatinus	XM_007666720	100077274	XP_007664910.1	tumor_protein_p73
Ursus_maritimus	XM_008687972	103660368	XP_008686194.1	cellular_tumor_antigen_p53
Ursus_maritimus	XM_008705162	103676025	XP_008703384.1	tumor_protein_63_isoform_X1
Ursus_maritimus	XM_008707805	103678460	XP_008706027.1	LOW_QUALITY_PROTEIN:_tumor_protein_p73
Peromyscus_maniculatus	XM_006973448	102922013	XP_006973510.1	cellular_tumor_antigen_p53
Peromyscus_maniculatus	XM_006984065	102904672	XP_006984127.1	tumor_protein_p73_isoform_X2
Peromyscus_maniculatus	XM_006985082	102922790	XP_006985144.1	tumor_protein_63_isoform_X1
Microtus_ochrogaster	XM_005344766	101985094	XP_005344823.1	tumor_protein_63_isoform_X1

Microtus_ochrogaster	XM_005349777	101978980	XP_005349834.1	cellular_tumor_antigen_p53
Microtus_ochrogaster	XM_005368637	101993208	XP_005368694.1	tumor_protein_p73_isoform_X3
Equus_przewalskii	XM_008516685	103548749	XP_008514907.1	tumor_protein_p73
Equus_przewalskii	XM_008530347	103557703	XP_008528569.1	cellular_tumor_antigen_p53
Pundamilia_nyererei	XM_005727159	102202371	XP_005727216.1	tumor_protein_63-like_isoform_X2
Pundamilia_nyererei	XM_005732992	102210912	XP_005733049.1	tumor_protein_p73-like_isoform_X1
Pan_paniscus	XM_008970761	100968381	XP_008969009.1	tumor_protein_p73_isoform_X6
Oryctolagus_cuniculus	NM_001082404	100009292	NP_001075873.1	cellular_tumor_antigen_p53
Oryctolagus_cuniculus	XM_002716509	100355881	XP_002716555.1	tumor_protein_63_isoform_X1
Oncorhynchus_mykiss	NM_001124692	100136737	NP_001118164.1	cellular_tumor_antigen_p53
Macaca_mulatta	XM_001092093	703997	XP_001092093.1	tumor_protein_63_isoform_4
Macaca_mulatta	NM_001047151	716170	NP_001040616.1	cellular_tumor_antigen_p53
Macaca_mulatta	XM_001083217	695164	XP_001083217.2	tumor_protein_p73-like_isoform_1
Acanthisitta_chloris	XM_009083033	103810144	XP_009081281.1	tumor_protein_p73_isoform_X1
Columba_livia	XM_005513215	102097847	XP_005513272.1	tumor_protein_63_isoform_X1
Columba_livia	XM_005514330	102090439	XP_005514387.1	tumor_protein_p73_isoform_X1
Falco_cherrug	XM_005437715	102059062	XP_005437772.1	tumor_protein_p73_isoform_X1
Falco_cherrug	XM_005443634	102049721	XP_005443691.1	tumor_protein_63_isoform_X1
Ovis_aries	NM_001009403	443421	NP_001009403.1	cellular_tumor_antigen_p53
Ovis_aries	XM_004003041	101108874	XP_004003090.1	tumor_protein_63_isoform_X1
Echinops_telfairi	XM_004705206	101647737	XP_004705263.1	tumor_protein_p73_isoform_X1
Echinops_telfairi	XM_004712913	101656530	XP_004712970.1	cellular_tumor_antigen_p53
Echinops_telfairi	XM_004713461	101638322	XP_004713518.1	tumor_protein_63
Otolemur_garnettii	XM_003791135	100963622	XP_003791183.1	cellular_tumor_antigen_p53_isoform_1
Otolemur_garnettii	XM_003792705	100953564	XP_003792753.1	tumor_protein_63_isoform_1
Otolemur_garnettii	XM_003793215	100941774	XP_003793263.1	tumor_protein_p73_isoform_1
Xiphophorus_maculatus	XM_005804434	102236502	XP_005804491.1	tumor_protein_63-like
Xiphophorus_maculatus	NM_001286289	102219062	NP_001273218.1	cellular_tumor_antigen_p53
Ceratotherium_simum	XM_004424577	101398344	XP_004424634.1	tumor_protein_63_isoform_1
Ceratotherium_simum	XM_004425512	101398093	XP_004425569.1	tumor_protein_p73_isoform_1
Ceratotherium_simum	XM_004433119	101399576	XP_004433176.1	cellular_tumor_antigen_p53
Physeter_catodon	XM_007100637	102985658	XP_007100699.1	cellular_tumor_antigen_p53
Physeter_catodon	XM_007103055	102996708	XP_007103117.1	tumor_protein_63_isoform_X4
Physeter_catodon	XM_007126048	102990708	XP_007126110.1	tumor_protein_p73
Lepisosteus_oculatus	XM_006627473	102690789	XP_006627536.1	cellular_tumor_antigen_p53-like
Lepisosteus_oculatus	XM_006637647	102698708	XP_006637710.1	tumor_protein_63-like_isoform_X1
Lepisosteus_oculatus	XM_006641954	102684765	XP_006642017.1	tumor_protein_p73-like_isoform_X1
Condylura_cristata	XM_004675090	101634665	XP_004675147.1	tumor_protein_63_isoform_X1
Condylura_cristata	XM_004684639	101632504	XP_004684696.1	cellular_tumor_antigen_p53_isoform_X1
Pongo_abelii	XM_002814408	100444448	XP_002814454.1	tumor_protein_63_isoform_X1
Pongo_abelii	XM_002826974	100435218	XP_002827020.1	cellular_tumor_antigen_p53
Galeopterus_variegatus	XM_008573838	103591400	XP_008572060.1	cellular_tumor_antigen_p53
Galeopterus_variegatus	XM_008574556	103591979	XP_008572778.1	tumor_protein_p73_isoform_X2
Galeopterus_variegatus	XM_008575976	103593057	XP_008574198.1	tumor_protein_63_isoform_X1
Sarcophilus_harrisii	XM_003768762	100922473	XP_003768810.1	cellular_tumor_antigen_p53_isoform_X1
Ictidomys_tridecemlineatus	XM_005330973	101966047	XP_005331030.1	tumor_protein_63_isoform_X1
Ictidomys_tridecemlineatus	XM_005332819	101957738	XP_005332876.1	cellular_tumor_antigen_p53
Ictidomys_tridecemlineatus	XM_005339216	101967531	XP_005339273.1	tumor_protein_p73_isoform_X1
Pseudopodoces_humilis	XM_005524985	102099399	XP_005525042.1	tumor_protein_63_isoform_X1
Pseudopodoces_humilis	XM_005528346	102101417	XP_005528403.1	tumor_protein_p73_isoform_X1
Pseudopodoces_humilis	XM_005533378	102101491	XP_005533435.1	cellular_tumor_antigen_p53-like
Cynoglossus_semilaevis	XM_008321788	103387239	XP_008320010.1	cellular_tumor_antigen_p53
Cynoglossus_semilaevis	XM_008338584	103399938	XP_008336806.1	tumor_protein_63_isoform_X1
Cynoglossus_semilaevis	XM_008317990	103384466	XP_008316212.1	LOW_QUALITY_PROTEIN:_tumor_protein_p73
Takifugu_rubripes	XM_003966884	101079595	XP_003966933.1	cellular_tumor_antigen_p53
Trichechus_manatus	XM_004376021	101351852	XP_004376078.1	cellular_tumor_antigen_p53
Trichechus_manatus	XM_004384309	101346253	XP_004384366.1	tumor_protein_p73_isoform_X1
Trichechus_manatus	XM_004382232	101349304	XP_004382289.1	tumor_protein_63_isoform_X1
Meleagris_gallopavo	XM_003212181	100545037	XP_003212229.2	tumor_protein_p73_isoform_X1
Nannospalax_galili	XM_008839677	103739334	XP_008837899.1	tumor_protein_63_isoform_X1
Nannospalax_galili	XM_008852667	103749787	XP_008850889.1	tumor_protein_p73_isoform_X4
Nannospalax_galili	XM_008855787	103752115	XP_008854009.1	cellular_tumor_antigen_p53
Bubalus_bubalis	XM_006053818	102398248	XP_006053880.1	tumor_protein_p73_isoform_X1
Bubalus_bubalis	XM_006060989	102398919	XP_006061051.1	tumor_protein_63_isoform_X1

Bubalus_bubalis	NM_001290844	102402069	NP_001277773.2	tumor_protein_p53
Leptonychotes_weddellii	XM_006738887	102726420	XP_006738950.1	cellular_tumor_antigen_p53
Leptonychotes_weddellii	XM_006748168	102744583	XP_006748231.1	tumor_protein_p73_isoform_X1
Xenopus_(Silurana)	NM_001001903	431679	NP_001001903.1	tumor_protein_p53
Xenopus_(Silurana)	XM_002934050	100497737	XP_002934096.2	tumor_protein_63_isoform_X1
Xenopus_(Silurana)	XM_002933855	100496308	XP_002933901.2	tumor_protein_p73
Erinaceus_europaeus	XM_007523372	103113788	XP_007523434.1	cellular_tumor_antigen_p53
Erinaceus_europaeus	XM_007525665	103115817	XP_007525727.1	tumor_protein_63_isoform_X1
Erinaceus_europaeus	XM_007527238	103117225	XP_007527300.1	tumor_protein_p73_isoform_X1
Gorilla_gorilla	XM_004024528	101138252	XP_004024577.1	tumor_protein_p73_isoform_1
Gorilla_gorilla	XM_004038198	101153691	XP_004038246.1	tumor_protein_63_isoform_2
Gorilla_gorilla	XM_004058511	101133524	XP_004058559.1	cellular_tumor_antigen_p53
Chrysemys_picta	XM_005279339	101942845	XP_005279396.1	cellular_tumor_antigen_p53
Chrysemys_picta	XM_005308059	101931345	XP_005308116.1	tumor_protein_63
Chrysemys_picta	XM_005298447	101939660	XP_005298504.1	tumor_protein_p73_isoform_X2
Zonotrichia_albicollis	XM_005484912	102073803	XP_005484969.1	tumor_protein_63_isoform_X1
Zonotrichia_albicollis	XM_005492626	102067209	XP_005492683.1	tumor_protein_p73_isoform_X3
Callithrix_jacchus	XM_002747948	100415710	XP_002747994.1	cellular_tumor_antigen_p53
Callithrix_jacchus	XM_002750200	100393670	XP_002750246.2	tumor_protein_p73
Callithrix_jacchus	XM_002758183	100386953	XP_002758229.1	tumor_protein_63_isoform_X1
Camelus_ferus	XM_006175816	102524476	XP_006175878.1	cellular_tumor_antigen_p53
Camelus_ferus	XM_006176713	102514514	XP_006176775.1	LOW_QUALITY_PROTEIN:_tumor_protein_p73
Camelus_ferus	XM_006188860	102515861	XP_006188922.1	tumor_protein_63_isoform_X1
Lipotes_vexillifer	XM_007457790	103089875	XP_007457852.1	cellular_tumor_antigen_p53_isoform_X1
Lipotes_vexillifer	XM_007448954	103073556	XP_007449016.1	tumor_protein_p73_isoform_X1
Taeniopygia_guttata	XM_002190889	100227936	XP_002190925.1	tumor_protein_63_isoform_X2
Maylandia_zebra	XM_004552828	101473710	XP_004552885.1	tumor_protein_63-like_isoform_X1
Maylandia_zebra	XM_004554110	101471388	XP_004554167.1	tumor_protein_p73-like_isoform_X3
Maylandia_zebra	XM_004571176	101480650	XP_004571233.1	cellular_tumor_antigen_p53-like_isoform_X1
Danio_rerio	NM_152986	260407	NP_694518.1	tumor_protein_63_isoform_alpha_1
Danio_rerio	NM_183340	368221	NP_899183.1	tumor_protein_p73
Danio_rerio	NM_001271820	30590	NP_001258749.1	cellular_tumor_antigen_p53_isoform_1

#Obsolete/Removed June 2015 (version 2014):

Organism	mRNAcodes	GeneIDs	ProteinCodes	Product
Taeniopygia_guttata	XM_002196500	100220605	XP_002196536.2	tumor_protein_p73_isoform_2
Pongo_abelii	XM_002811581	100433100	XP_002811627.1	tumor_protein_p73
Meleagris_gallopavo	XM_003209131	100549371	XP_003209179.1	tumor_protein_63-like
Nomascus_leucogenys	XM_003274633	100583326	XP_003274681.2	LOW_QUALITY_PROTEIN:_cellular_tumor_antigen_p5
Pan_troglodytes	XM_003307781	100611345	XP_003307829.1	LOW_QUALITY_PROTEIN:_tumor_protein_p73
Saimiri_boliviensis	XM_003927009	101037700	XP_003927058.1	tumor_protein_63_isoform_2
Saimiri_boliviensis	XM_003939632	101036272	XP_003939681.1	tumor_protein_p73
Takifugu_rubripes	XM_003963137	101074693	XP_003963186.1	tumor_protein_p73-like_isoform_1
Takifugu_rubripes	XM_003973878	101080154	XP_003973927.1	tumor_protein_63-like
Ovis_aries	XM_004014035	101118974	XP_004014084.1	LOW_QUALITY_PROTEIN:_tumor_protein_p73
Oryzias_latipes	XM_004068304	100170632	XP_004068352.1	tumor_protein_63
Dasypus_novemcinctus	XM_004468752	101416069	XP_004468809.1	cellular_tumor_antigen_p53_isoform_3
Dasypus_novemcinctus	XM_004469613	101418539	XP_004469670.1	tumor_protein_63_isoform_1

#NR database:

ProtCode	mRNAcode	range	Description
p53_AEW46988	JN794073	58-1221	Callorhinchus_milii_tumor_protein_p53
p63_AEW46989	JN794074	64-2151	Callorhinchus_milii_tumor_protein_p63
