# Supplementary material for: Functional Diversification after Gene Duplication: Paralog Specific Regions of Structural Disorder and Phosphorylation in p53, p63, and p73
Source: PLoS One. 2016 Mar 22;11(3):e0151961. doi: 10.1371/journal.pone.0151961 (PMC4803236; doi:10.1371/journal.pone.0151961)
Supplement: S2 Table — (PDF) [file pone.0151961.s011.pdf]

Supplementary material

S2 Table PDB files and regions used for mapping DOT and disorder conservation into a structural context

S2 Table.

Pfam Domain	PDB code	Protein template	PDB numeration	Sequence positions (human)	Fragment length	Alignment sites
TAD_p53	3dac	p53	17-28	17-28	12	231-242
TAD_p63	3dac	p53	17-28	53-64	12	231-242
TAD_p73	3dac	p53	17-28	13-24	12	231-242
DBD_p53	4hje	p53	94-291	94-291	198	410-660
DBD_p63	4hje	p53	94-291	162-361	198	410-660
DBD_p73	4hje	p53	94-291	112-311	198	410-660
OD_p53	1olg	p53	319-360	319-360	42	717-768
OD_p63	4a9z	p63	358-404	397-443	47	731-802
OD_p73	4a9z	p63	358-404	351-392	47	731-802
