# Supplementary material for: Functional Diversification after Gene Duplication: Paralog Specific Regions of Structural Disorder and Phosphorylation in p53, p63, and p73
Source: PLoS One. 2016 Mar 22;11(3):e0151961. doi: 10.1371/journal.pone.0151961 (PMC4803236; doi:10.1371/journal.pone.0151961)
Supplement: S3 Table — Alignment sites following a 50% majority rule of sequences with phosphorylation predictions based on NetPhos phosphorylation prediction score cut-off = 0.75 (gaps included). Information displayed per clade (specific) and per family (shared). Shaded areas correspond to the majority rule (phosphorylation predicted for more than 50% of taxa per clade or for the family). Corresponding positions in the canonical human proteins (P53_human NP_000537.3, P63_human NP_003713.3, and P73_human NP_005418.1) are shown. (PDF) [file pone.0151961.s012.pdf]

Supplementary material

S3 Table Shared and clade specific predicted phosphorylation patterns. Alignment sites following a 50% majority rule of sequences with phosphorylation predictions based on NetPhos phosphorylation prediction score cut-off=0.75 (gaps included). Information displayed per clade (specific) and per family (shared). Shaded areas correspond to the majority rule (phosphorylation predicted for more than 50% of taxa per clade or for the family). Corresponding positions in the canonical human proteins (P53_human NP_000537.3, P63_human NP_003713.3, and P73_human NP_005418.1) are shown.

S3 Table.				
p53 clade-specific sites	Domain	p53_human	p63_human	p73_human
432	DBD	116	184	134
471	DBD	155	223	173
539	DBD	211	281	231
653	DBD	284	354	304
681	Linker2	303	373	325
706	Linker2	315	385	337
796	Linker3	366	439	392
803	Linker3	367	444	393
809	Linker3	371	448	397
1088	Cter	376	652	596
1091	Cter	378	654	598
1156	Cter	392	679	635
p63 clade-specific sites		p53_human	p63_human	p73_human
149	Nter	-	25	-
154	Nter	-	30	-
163	Nter	-	34	-
164	Nter	-	35	-
324	Linker1	-	111	65
362	Linker1	-	131	82
386	Linker1	-	142	-
405	Linker1	92	160	110
444	DBD	128	196	146
498	DBD	182	250	200
657	DBD	288	358	308

673	Linker2	297	367	S319
712	Linker2	-	389	343
720	OD	322	395	349
744	OD	337	410	364
833	Linker3	-	S452	401
839	Linker3	-	458	406
840	Linker3	-	459	407
844	Linker3	-	463	411
862	Linker3	-	477	426
947	Linker3	-	535	479
976	SAM	-	564	508
992	SAM	-	580	524
998	SAM	-	586	530
1058	Cter	-	627	570
1065	Cter	-	630	573
1067	Cter	-	631	575
1081	Cter	-	645	589
**p73 clade-specific sites**		**p53_human**	**p63_human**	**p73_human**
253	TAD	30	74	26
255	TAD	32	76	28
332	Linker1	56	119	70
361	Linker1	-	130	81
464	DBD	148	216	166
658	DBD	289	359	309
696	Linker2	311	381	333
735	OD	328	401	355
754	OD	347	420	374

**1066**	**Cter**	**-**	**-**	**574**
**1130**	**Cter**	**380**	**670**	**621**
**1136**	**Cter**	**384**	**674**	**625**
**Overlapping sites across paralogues**		**p53_human**	**p63_human**	**p73_human**
**225**	**TAD**	**15**	**51**	**11**
**364**	**Linker1**	**70**	**132**	**83**
**392**	**Linker1**	**81**	**147**	**97**
**399**	**Linker1**	**87**	**154**	**104**
**415**	**DBD**	**99**	**167**	**117**
**419**	**DBD**	**103**	**171**	**121**
**434**	**DBD**	**118**	**186**	**136**
**437**	**DBD**	**121**	**189**	**139**
**533**	**DBD**	**205**	**275**	**225**
**543**	**DBD**	**215**	**285**	**235**
**638**	**DBD**	**269**	**339**	**289**
**689**	**Linker2**	**304**	**374**	**326**
**736**	**OD**	**329**	**402**	**356**
**770**	**OD**	**361**	**434**	**388**
